# Bone Healing Monitoring in Bone Lengthening Using Bioimpedance

**DOI:** 10.1155/2022/3226440

**Published:** 2022-04-07

**Authors:** Farahnaz Sadoughi, Ali Behmanesh, Farid Najd Mazhar, Mohammad Taghi Joghataei, Shahram Yazdani, Roshanak Shams, Hassan Morovvati, Sareh Najaf Asaadi, Araz Vosough

**Affiliations:** ^1^Health Management and Economics Research Center, School of Health Management and Information Sciences, Iran University of Medical Sciences, Tehran, Iran; ^2^Bone and Joint Reconstruction Research Center, Department of Orthopedics, School of Medicine, Iran University of Medical Sciences, Tehran, Iran; ^3^Education and Development Center, Iran University of Medical Sciences, Tehran, Iran; ^4^Cellular and Molecular Research Center, Iran University of Medical Sciences, Tehran, Iran; ^5^School of Management & Medical Education Sciences Shahid Beheshti University of Medical Sciences, Tehran, Iran; ^6^Department of Basic Science, Faculty of Veterinary Medicine, University of Tehran, Tehran, Iran; ^7^Department of Clinical Sciences, Faculty of Veterinary Medicine, Garmsar Branch, Islamic Azad University, Garmsar, Iran

## Abstract

The most common technique of orthopedic surgical procedure for the correction of deformities is bone lengthening by “distraction osteogenesis,” which requires periodic and ongoing bone assessment following surgery. Bone impedance is a noninvasive, quantitative method of assessing bone fracture healing. The purpose of this study was to monitor bone healing and determine when fixation devices should be removed. The left tibia of eight male New Zealand white rabbits (2.4 ± 0.4 kg) undergoing osteotomy was attached with a mini-external fixator. The bone length was increased by 1 cm one week after surgery by distracting it 1 mm per day. Before and after osteotomy, as well as every week after, bone impedance was measured in seven frequency ranges using an EVAL-AD5933EBZ board. Three orthopedic surgeons analyzed the radiographs using the Radiographic Union Scale for Tibial (RUST) score. The Kappa Fleiss coefficient was used to determine surgeon agreement, and the Spearman rank correlation coefficient was used to find out the relationship between impedance measurements and RUST scores. Finally, the device removal time was calculated by comparing the bone impedance to the preosteotomy impedance. The agreement of three orthopedic surgeons on radiographs had a Fleiss' Kappa coefficient of 49%, indicating a moderate level of agreement. The Spearman rank correlation coefficient was 0.43, indicating that impedance and radiographic techniques have a direct relationship. Impedance is expected to be used to monitor fractured or lengthened bones in a noninvasive, low-cost, portable, and straightforward manner. Furthermore, when used in conjunction with other qualitative methods such as radiography, impedance can be useful in determining the precise time of device removal.

## 1. Introduction

“Distraction Osteogenesis” is a typical treatment for bone deformities and limb length discrepancy. Throughout the previous few decades, this surgery has been performed to address dwarfism or skeletal deformities caused by congenital defects, traumas, tumors, or infections. However, the discrepancy between the limbs, particularly the long bones like the femur and tibia, causes wear and tear on the knee joint as well as spine curvature, compromising the patient's health and quality of life [1]. The phases of distraction osteogenesis are as follows [2]:

1. Osteotomy: Bone is split into two parts during this phase.
2. Latency period: This period occurs after osteotomy and is characterized by the formation of hematomas at the site of the osteotomy, which are later replaced by granulation tissue. Immature bone (callus) forms during this period. It usually takes 5 to 10 days for this to happen.
3. Distraction: Throughout this phase, the fixation device gradually applies a tensile force to both segments of bones at a rate of one millimeter every day.
4. Consolidation: After the desired length of bone has been achieved, this phase begins. At this stage, the fixation device remains on the bone to form a hard callus within the gap between the bone segments, the collagen bundles become calcified, and osteoblasts appear.
5. Device removal: The removal time is determined by radiography and medical examination, and the fixation device is separated from the bone at this time. This is the final phase of the bone lengthening process.

The diagnosis of a fully healed bone fracture is critical for making the best management decisions, but various studies have revealed that there is no standard diagnostic technique in this field [3–5]. Radiography and physical examination are two popular approaches for monitoring the healing process of bone fractures used in medical centers [6]. Radiographs are taken during the beginning of bone lengthening, the end of the distraction phase, and the beginning of bone union (consolidation or device removal phases). Some studies, however, have found that radiography alone cannot adequately depict fracture healing and is unreliable in determining the stage of fracture repair [7]. X-ray radiography is a common approach for identifying bone fractures that exposes a patient to higher quantities of radiation, especially if the patient must be examined multiple times. These tests are extremely harmful to children, pregnant women, and the elderly, not to mention quite expensive [6, 8, 9]. Computed Tomography (CT), Dual Energy X-ray Absorptiometry (DXA/DEXA), and ultrasound can all be used to diagnose bone fractures, but their use in medical centers is limited due to their high cost and radiation exposure [7, 10]. In addition, physical examinations by a physician are frequently undertaken to check the fracture more closely. Physician examinations are subjective, which might result in varying and erroneous judgments [3].

There are various quantitative techniques for evaluating bone fractures healing process, such as ultrasound [11], bending stiffness test [12], measurement of bone mineral density [13]^,^ quantitative computed tomography (QCT) [14], acoustic emission [15], and vibratory devices [16]. Many of these techniques are either not available at every healthcare center or are restricted in number and must be reserved in advance. Furthermore, these tests cannot be performed at the patient's home, and the patient must be transferred to the device location, which is both difficult and uncomfortable for the patient [6].

Nowadays, monitoring the fractures healing process is one of medicine's most challenging tasks and hot research issues. As a result, some researchers focused on mechanical bone feedback [17, 18], while others used electrical techniques to evaluate fractures [19]. Much research on the bone structure has been done in the last century, leading to the discovery of several intriguing properties in bone. Some of these research studies revealed that bone exhibits dielectric and semiconductor properties, while others revealed anisotropic and piezoelectric capabilities [20]. Several studies have found an association between fracture healing and bone electrical impedance [21, 22]. As a result, bone's electrical impedance can be employed as a quantifiable and noninvasive measurement tool for monitoring bone fractures [9, 21].

For bone lengthening, unilateral or ring external fixation devices are commonly employed and must be implanted and carried on the bone for several months. Traditionally, orthopedic surgeons determined the time of fixation device removal from bone by examining the patient and taking radiographs [21]. Because some bone assessment methods are subjective, costly, and inaccessible to patients, the goal of this study is to propose a low-cost, noninvasive alternative for bone healing monitoring and determining when fixation devices should be removed from the bone.

## 2. Material and Methods

**Figure 1:**
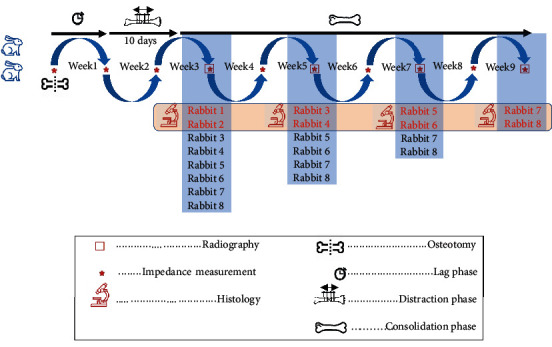
Flow diagram of the study: one week after osteotomy, the two bone segments were distracted for 10 days (1 mm per day). The bone's electrical impedance was measured three times (before, after, and every week following osteotomy). Weekly radiographs and electrical impedance tests were done after the distraction. It depicts the process flow of the current study.

The approval of the National Committee on Ethics in Biomedical Research of Iran University of Medical Sciences (ethic code: “IR.IUMS.REC.1397.852”) was obtained. All experiments were performed in accordance with relevant guidelines and regulations, and this study adheres to ARRIVE Guidelines for reporting animal research [23]. The experimental study design is depicted in Figure 1.

### 2.1. Animals and Surgical Procedures

Eight male New Zealand white rabbits (2.4 ± 0.4 kg) have been used in this experiment. These rabbits were divided into four groups of two (weeks 3, 5, 7, and 9 after osteotomy). Rabbits in the first to fourth groups were sacrificed for bone histology at the third, fifth, seventh, and ninth weeks after osteotomy. Rabbit housing and surgery were carried out at Iran University of Medical Sciences' Center for Experimental and Comparative Studies Center in accordance with the Islamic Republic of Iran's Ministry of Health's ethical guidelines for animal research.

**Figure 2:**
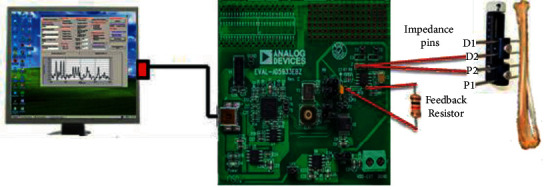
Configuration AD5933 evaluation board.

These surgeries were carried out by a board-certified orthopedic surgeon. Under intramuscular anesthesia with ketamine (50 mg/kg) and xylazine (10 mg/kg), two 10 mm skin incisions were made: one at the half end of the rabbit's left tibia (the fibula-tibia junction) and one 15 mm away at the distal end of the left tibia. In this study, an aluminum mini-external fixator (60 mm length) and four stainless-steel pins (2 mm diameter) were employed to lengthen the bone. It was surgically attached to the rabbits' left tibia. Two threaded, stainless-steel pins (2.0 mm diameter) were inserted perpendicular to the bone axis at the P1 and D1 sites (Figure 2). Then, a unilateral external fixator was placed on them. Similarly, the remaining two pins were inserted into the bone at P2 and D2. The pins were insulated before attaching the fixator's clamp to them. Between the P2 and D2 pins, a 10 mm skin incision was made, the skin was peeled away to expose the periosteum, and osteotomy was performed with an orthopedic saw. The surgical wound was closed with sutures. Following surgery, Vetafluxin (Flunixin meglumine) was injected intramuscularly to relieve pain, inflammation, and fever, and an antibiotic, Enrofloxacin, was injected intramuscularly.

### 2.2. Measurement of Bone Electrical Impedance

**Figure 3:**
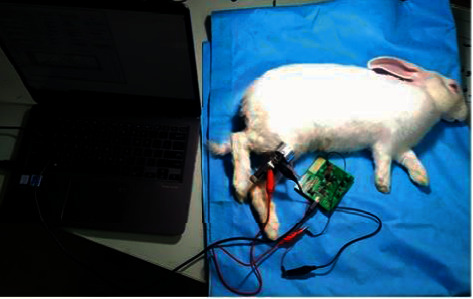
Measurement of rabbit bone impedance.

The Analog Device company's EVAL-AD5933EBZ board was used to measure the impedance of the bone. This board's measurements were transmitted to the PC through a USB cable. (See Figures 2 and 3). Before measuring the bone impedance, two 1 ohm resistors were connected to the board's “unknown impedance” and “feedback resistor” input pins, and the board was calibrated. The resistor connected to “unknown impedance” was then removed, and pins (D2 and P2) were connected in their places (Figure 2). Bone impedance was measured at three different times: (1) before the osteotomy, (2) just after osteotomy, and (3) every week after the osteotomy. To avoid any negative impacts on impedance measurements, rabbits were anesthetized with ketamine injections (Figure 3). Impedance was evaluated in seven frequency ranges (10-1 k, 1 k-2 k, 2 k–4 k, 4 k–8 k, 8 k–16 k, 16 k–32 k, and 32 k–51.2 k Hz) to discover the electrical impedance behavior of bone.

Because rabbit impedance values differ, the impedance values of the bone were standardized using the following formula.

(1) $$\begin{array}{c} \mathrm{Impedance}\;\mathrm{normalized=}\frac{\mathrm{impedance}}{\mathrm{impedance}\;\mathrm{in}\;\mathrm{first}\;\mathrm{week}}. \end{array}$$

The bone impedance graphs and mean values of each rabbit at different frequencies were used to assess the trend of changes in bone impedance over time.

### 2.3. Radiographic Examination

After the distraction phase was completed, radiographic examinations were taken every week (third week onwards) at a 50 kV effective voltage and a 100 mA current. Due to the existence of a fixation device on the bone, radiographic examinations were taken at two views, oblique and anterior-posterior. To assess tibial healing in the radiographic examination, the Radiographic Union Scale for Tibial (RUST) fracture was used as a novel fracture assessment tool. The union of bone after the osteotomy is scored by RUST [19, 24]. Three blinded orthopedic surgeons from the Iran University of Medical Sciences' Shafa Yahyaian Educational and Medical Center assigned a RUST score to each of the 25 sets of radiographs (oblique and anterior-posterior). We sent these radiographs in RUST form (available in Table A in the Supplementary Material section) to three surgeons independently. In Table A, we have used the RUST score as a quantitative fracture assessment tool to help us quantify the radiographic assessment of tibial fractures. Orthopedic surgeons evaluate 25 pairs of radiographs (anterior-posterior and oblique) to mark the appropriate areas on the RUST scoring form. Only one of the four RUST criteria in the right or left cortex of the bone is marked on anteroposterior or oblique radiographs. The radiographs of eight rabbits are taken at different weeks following osteotomy. In the table's left column, the rabbit's number and the number of weeks of radiography following osteotomy are listed.

**Figure 4:**
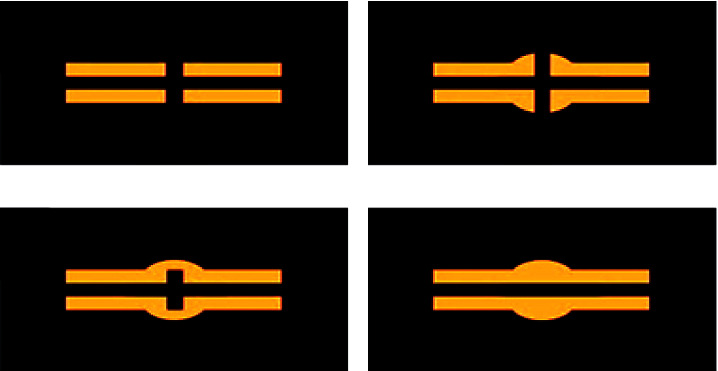
Scoring of radiograph using RUST: (a) the presence of a fracture line with no callus formation (this is scored as 1), (b) the presence of a fracture line with soft callus formation (this is scored as 2), (c) bridging callus (this is scored as 3), and (d) complete bridging of the callus without a fracture line (this is scored as 4).

The surgeons were not given any information about the rabbits in order to avoid bias.  Figure 4 explains how to use the RUST to score radiographs. Three orthopedic surgeons scored the two cortices in each of the oblique and anterior-posterior views (in total four cortices). Based on the sum of the RUST scores for the four bone cortices in the radiographs (between 4 and 16), the radiographs are divided into three categories: “No Union” (NU = between 4 and 9), “Suspect” (SUS = between 10 and 11), and “Union” (UN = between 12 and 16).

The Fleiss' Kappa coefficient was used to evaluate the reliability of agreement among three surgeons [25].  Table 1 interprets interrater reliability (IRR). The Spearman rank correlation coefficient was used to discover the strength of a link between impedance and radiography.

Finally, the radiographs were categorized into three general classes (UN, NU, and SUS) as follows: if at least two of the three surgeons determine that the bone is union or nonunion, the radiograph is classified as UN or NU. Due to the sensitivity of fracture union diagnosis, suspected cases are classified as follows; if two surgeons' opinions are suspicious and one surgeon's opinion is union or nonunion, the final class is SUS or NU. As a result, values greater than 11.5 are classified as UN.

### 2.4. Histology

For tissue study, the left tibia bones were removed and, in 10% formalin, transported to the University of Tehran's Faculty of Veterinary Medicine's Histology Department. The samples' decalcification was done with 5% nitric acid solution. Then, tissue processing (dehydration, clarification, and paraffin) was performed, and the samples were molded. Sections of 7 *μ*m thick were prepared from tissue molds and stained with hematoxylin and eosin. Eventually, tissue sections were imaged with a light microscope.

### 2.5. Determination of Device Removal Time

In the bone impedance measurement approach, linear regression analysis was performed to determine the time of device removal from the bone. First, we calculated the “cut-off threshold” for each bone impedance graph. Passing the graph of bone impedance through the cut-off threshold (impedance in preosteotomy) indicates the time of removing the device.

### 2.6. Data Analytics Tools

The Fleiss' Kappa coefficient, Spearman rank correlation coefficient, and linear regression analysis were all performed using the R programming language (ver 3.5.3) and the RStudio Integrated Development Environment (ver. 1.2.5033). In addition, several graphs were created using Microsoft Excel (2019).

## 3. Results

### 3.1. Bone Impedance

**Figure 5:**
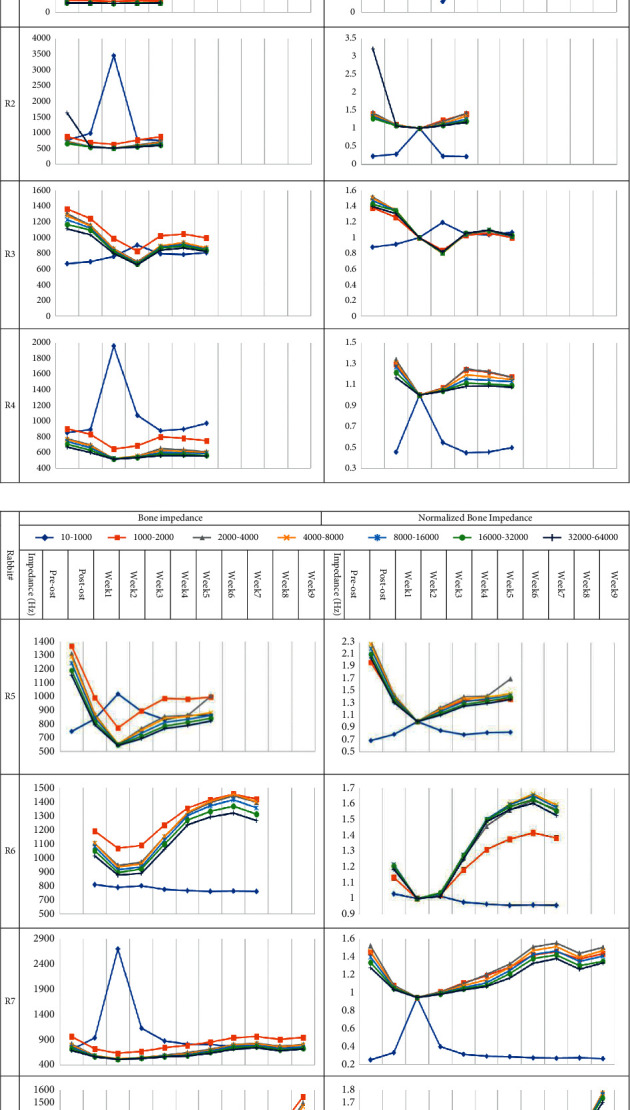
Bone impedance graph (left) and normalized bone impedance graph (right) for eight rabbits.

Figure 5 illustrates bone impedance values in seven frequency ranges for eight rabbits, demonstrating that the range of impedance values differs for rabbits. To compare these plots, the normalized impedance values for each rabbit were calculated and plotted. Each graph demonstrates that the process of changing impedance is the same for frequencies greater than 1 kHz.

Before and after osteotomy, there was a statistically significant difference in bone impedance (*p* value = 0.001). Prior to the first week at the inflammation phase, impedance was decreased, and after that, at the bone distraction phase, impedance was increased. In other words, the impedance is the lowest during the first week. However, because of the loosening of the pin, the results differ in rabbit 2. When the normalized impedance values are compared, it is clear that the bone impedance in the frequency range of 10–1000 Hz is at the bottom of the graph, while the impedance values in the other frequency ranges are at the top.

**Figure 6:**
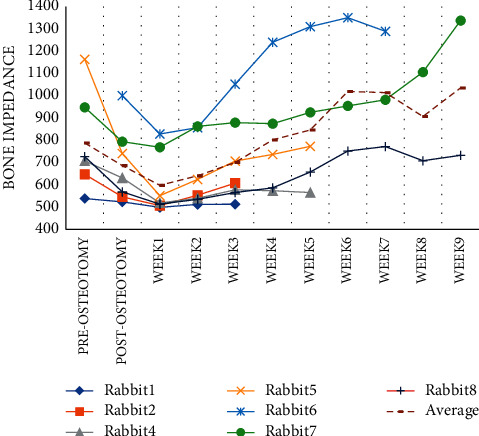
The average impedance over the frequency range of 16000–32000 Hz.

**Figure 7:**
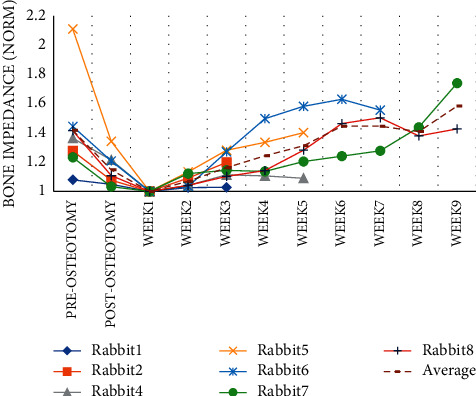
The average normalized impedance over the frequency range of 16000–32000 Hz.

The frequency range of 16 k–32 k Hz was analyzed as a sample due to the comparable pattern noticed in bone impedance in different weeks. The averaged impedance of all rabbits in this frequency range was computed and shown as a dashed line. Figures 6 and 7 reveal that the impedance increased from the first to the sixth week and reduced in the seventh and eighth weeks.

### 3.2. Radiography

Table 2 shows bone impedance measurements, Radiographic Union Scale for Tibial (RUST) scores of three orthopedic surgeons, and bone union status classification based on RUST scores. The Fleiss' Kappa coefficient for agreement among the three surgeons was 0.49 (*p* value 0.001). This coefficient has a value ranging from 0.41 to 0.60, indicating “moderate agreement.” The results of this section demonstrate that the RUST score for the rabbit-week twins (R4-W4), (R6-W5), (R3-W6), (R6-W6), (R6-W7), (R8-W8), (R7-W9), and (R8 -W9) was greater than 11.5, indicating that bone healing was detected by radiography at least in the fourth week.

**Figure 8:**
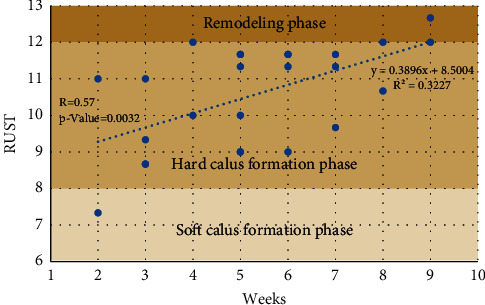
The linear regression of normalized impedance and RUST scores.

Figure 8 depicts the stages of bone formation based on RUST scores over the course of several weeks. This graph depicts the formation of a soft callus in the second week, a hard callus in the third to eighth weeks, and bone remodeling in the ninth week. The Spearman rank correlation coefficient between RUST and weeks is 0.57 (*p* value 0.05), demonstrating a significant direct association between RUST and weeks.

### 3.3. Histology

Group 1: In the microscopic study, at the site of a bone fracture, the cartilage tissue was observed along with chondroblast and chondrocytes cells, which is a stage of bone repair after a fracture. Osteogenic periosteum cells and endosteum participate in the formation of the callus. The cells close to bone crumbs became bone fragments in the presence of blood vessels, and the cells that are far from the proliferation area and blood vessels transformed into chondroblast and form external callus cartilage. In general, cartilage is a temporary fracture and bone replaces it.

**Figure 9:**
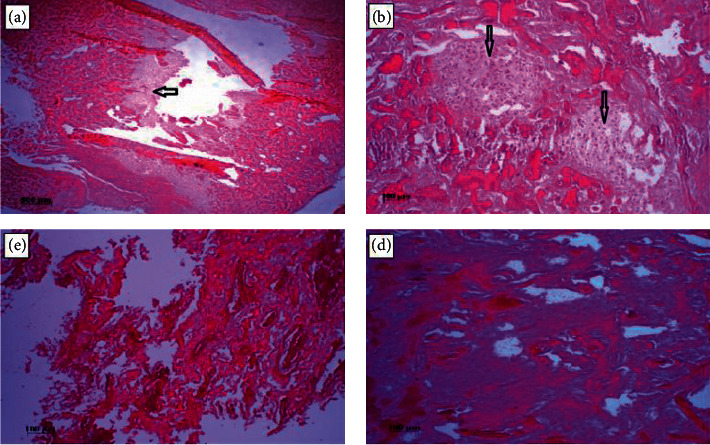
Tissue image of group 1 rabbit tibia bone in which cartilage can be seen (a), cartilage tissue (black arrow) can be seen as bone tissue (b), bone lamellae are formed and no cartilage tissue is seen (c), and the bony lamellae are well formed at the site of the bone fracture (d).

In the microscopic examination of group 1, external callus cartilage formation was observed. But according to the radiology report, which showed that in group 1 no bridge was formed and there was a gap resulting from the fracture. It should be noted that cartilages transmit X-rays in radiology imaging and therefore were not observed in the imaging (Figure 9(a)). Group 2: microscopic examination showed that a large percentage of formed cartilage as external callus transmitted bony lamellae. Therefore, it is reported in radiology images the callus bridge indicated the formation of bony lamellae (Figure 9(b)). Group 3: fully bony lamellae were observed at the fracture site (Figure 9(c)). Group 4: bone density was higher in the lamellae which showed complete repair of tibia bone tissue. Radiology reports in groups 3 and 4 confirmed the same results (Figure 9(d)).

### 3.4. Impedance versus Radiography

**Figure 10:**
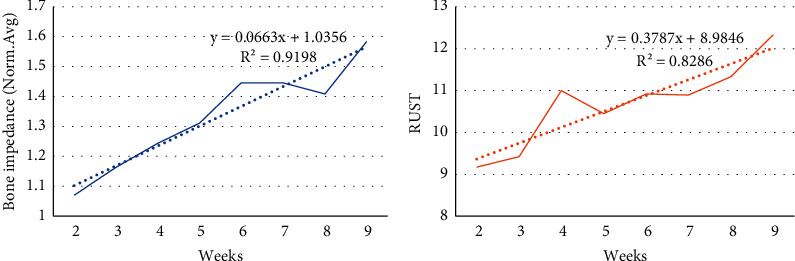
Normalized impedance changes over time (a). RUST score changes over time (b).

The average value of impedance over weeks is depicted in Figure 10(a), whereas the average value of RUST scores is depicted in Figure 10(b). These graphs show an increase in impedance and RUST score over the course of several weeks.

**Figure 11:**
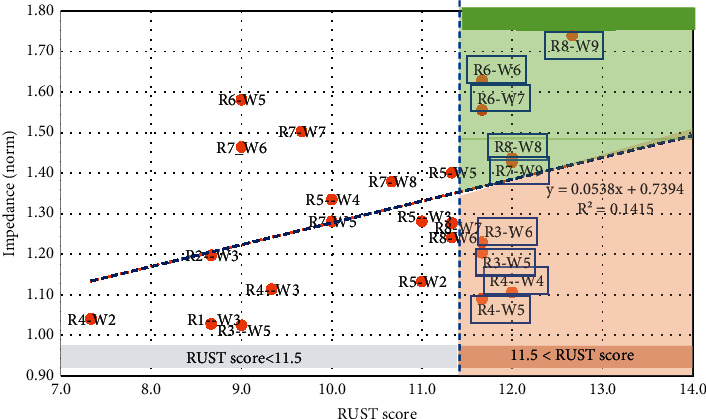
The relationship between rabbit bone impedance and RUST scores.

Figure 11 depicts the relationship between impedance measurements and RUST scores in similar weeks. The Spearman rank correlation coefficient was found to be 0.43 (*p* value 0.05), indicating a direct and substantial association between impedance and radiography approaches. RUST scores of tree rabbits 4, 7, and 8 were more than 12. But the bone impedance of two rabbits, 7 and 8, was above the regression line.

RUST scores greater than 11.5 in the graph (right section) show that the bone has been consolidated. As a result of radiography, rabbits 3, 4, 6, 7, and 8's bone fractures have healed. The bone is healed if the impedance and RUST score are in the top-right corner of this graph. In this area, impedance and RUST scores are substantially higher, indicating bone healing. RUST scores for rabbits 3 and 4 are high, but impedance is low. The bottom-right corner is the challenging area that requires more examination. As a result, the healing of bone fractures in rabbits 6, 7, and 8 was detected using two bone fracture assessment methods: radiography and electrical impedance.

**Figure 12:**
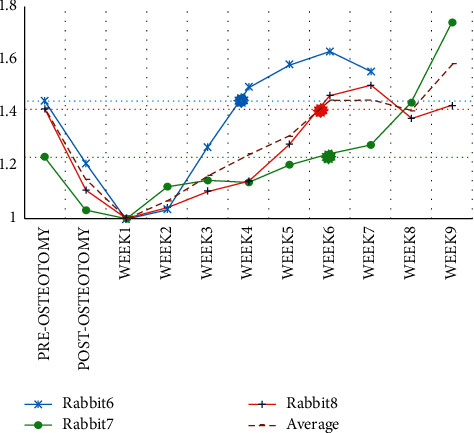
The cut-off threshold for removing the fixation device from the bones of rabbits 6, 7, and 8.

In the weeks following osteotomy, only rabbits 6, 7, and 8's impedances were higher than their preosteotomy impedances.  Figure 12 depicts the normalized impedance of these three rabbits, as well as their cut-off threshold. Crossing the normalized impedance graph of each rabbit's bone beyond the cut-off threshold of the rabbit graph reveals that the bone is as strong as it was before the osteotomy. As a result, rabbit 6's bones healed at week 4 while rabbits 7 and 8's bones healed at week 6.

## 4. Discussion

The current work aimed to provide a low-cost, noninvasive, and portable method of monitoring bone healing in order to minimize patient travel and high-risk experiments such as radiography; thus, this approach should be capable of sensing, analyzing, and transmitting bone status data. As a result, bone impedance measurement was employed in the current work to detect bone status and provide quantitative data in order to monitor bone lengthening.

Various electrical impedance meters have been utilized in various research to date. Lin et al. developed miniature sensors/electrodes that were put into the fracture site and connected to the Agilent E4980AL Precision LCR Meter to determine the electrical impedance of the animal's bone [19]. In other studies, researchers drilled a pair of stainless-steel electrodes into the bone [6, 21, 26] and measured bone impedance with such an AD5933 [6, 27] or a laboratory-designed impedance circuit [21, 26]. Yoshida's et al. study was the only one in which the impedance approach was utilized to assess lengthened bone in experimental rabbits [21]. In other studies, heavy and noncarriable instruments were used to assess bone fractures using the impedance approach. As a result, due to their large size and weight, researchers' desktop impedance meters are not suited for portable monitoring. The AD5933, which is about the size of a credit card, is less expensive than other portable impedance meters and can sweep the frequency quickly (1 Msps sampling speed) [28]. As a result, the AD5933 evaluation board was employed to evaluate impedance in this study. Measurements in several frequency ranges revealed that while this board lacks the accuracy required in the frequency range of 10–1000 Hz, it works correctly in all other frequency ranges above 1000 Hz.

Numerous studies have demonstrated that having water in the tissue increases the conductivity. As a result, impedance values in tissues with less water, such as bone, are greater than impedance values in tissues with a lot of water, such as blood and muscle [29, 30]. Because bleeding at the incision site causes inflammation following an osteotomy, impedance value falls during the inflammatory phase [19, 31]. In line with these findings, the findings revealed that the impedance value was the lowest in the first week following osteotomy for two reasons. First, the incised area was irritated for a week prior to the first week, such that the inflammation subsided after one week and callus gradually formed. Second, during the distraction phase, the bone length between the two impedance electrodes grows by one millimeter per day. Because growing body length increases impedance [9], the quantity of impedance of the bone increased during bone lengthening.

In line with our findings, Yoshida et al. discovered that the amount of bone impedance grew throughout the trend of fracture healing, with only a tiny decrease in the fourth and fifth weeks [21]. The current study's results of measuring bone impedance, together with the findings of prior studies, demonstrated that when bone healed, the amount of bone impedance rose. The average impedance curve demonstrated a decrease in bone impedance in the seventh and eighth weeks.

Several studies in this field using diverse experiments such as histology, radiography, and micro CT revealed a direct association between bone healing and impedance [21, 26, 32]. Lin et al. assessed bone fracture healing using RUST and impedance methods [19]. To date, no study on bone lengthening has used the RUST score to evaluate lengthened bone. We used RUST as well as the impedance approach in this study to evaluate the healing process of the lengthened bone.

The findings revealed a moderate relationship between the two methods of impedance and radiography. In general, determining the optimal time for fracture healing is critical for fixator removal. However, because it is difficult to determine when to remove the fixator from the bone [28], decisions based on radiography and clinical examination have poor interobserver and intraobserver reliability. If the device is removed too soon, it will cause refracture or gradual bone tilting. According to reports, fractures during healing range from 3% to 50% [33]. Studies demonstrated that the rate of fractures after fixator removal was reduced by 3.6 percent when Osteodensitometry was used [34]. Aside from movement limitations, the device's prolonged presence on the bone may have a negative social and psychological impact on patients [35]. Zhao et al. used radiography to determine the presence of a full cortex at the lengthened site [36]. Saran et al. used DEXA in a study to determine when to remove the device from patients' bones. They decided that the fixator was removed when bone mineral density increased by at least 10% [33].

According to Djasim et al., the rabbit fractured bone heals in 14 to 56 days [35]. The normal distribution for this time period indicates that 35 days (five weeks) is sufficient for healing. According to the findings of this study, the lengthened bone will take six weeks to heal. Our results showed that the RUST scores of rabbits 4, 7, and 8 in the fourth, eighth, and ninth weeks were greater than 12, indicating that it was time to remove the device, according to the radiography findings.

The bone impedance measurements of these rabbits, on the other hand, showed that the normalized impedance of rabbits 8 and 7 was 1.43 and 1.74 in the ninth and eighth weeks, respectively, showing that the fractures were fully healed. In the fourth week, the normalized impedance of rabbit 4 was 1.11. The rabbit 4 impedance graph revealed that the impedance began to decline after the third week, and the normalized value of this rabbit impedance in the fourth week had not changed considerably from the first week. In contrast to the impedance approach, radiography shows bone healing in this rabbit in the fourth week, based on RUST scores and impedance measurements.

Surgeons' interpretations of radiographs are qualitative and subjective, surgeons have different perspectives and perceptions of radiographs, and it appears that radiography alone is not a suitable method for determining the time of device separation from bone. The findings of our study are consistent with the findings of another study, which found that radiography could not accurately describe bone union and was unreliable in determining the stage of fracture healing [7]. It should be highlighted that the impedance approach can support surgeons in making the right decision.

## 5. Conclusion

Impedance is expected to be used to monitor fractured or lengthened bones in a noninvasive, low-cost, portable, and straightforward manner. Impedance can be useful in monitoring bone and determining the precise time of device removal when combined with other qualitative methods such as radiography. More research with a larger number of animal models, followed by human subjects, is needed to validate these findings. Because musculoskeletal conditions and their consequences are common, providing services to all orthopedic patients has limitations, such as physician referrals and frequent examinations, which can be costly for both the patient and the healthcare system. The bone impedance measurement is the preferred method for bone healing monitoring. The use of the impedance technique in bone monitoring is expected to decrease the number of radiographs taken and, as a result, the number of referrals to the surgeon, hence reducing overall healthcare costs.

## Figures and Tables

**Table 1:** Interpretation of Fleiss' Kappa coefficient [25].

Fleiss' Kappa	Interpretation

<0	Poor agreement
0.01–0.20	Slight agreement
0.21–0.40	Fair agreement
0.41–0.60	Moderate agreement
0.61–0.80	Substantial agreement
0.81–1.00	Almost perfect agreement

**Table 2:** RUST scores of three orthopedic surgeons.

Radiograph #	Rabbit #	Weeks after osteotomy #	Normalized impedance	Cortex O-R			Cortex O-L			Cortex AP-R			Cortex AP-L			Total			Mean	Classes			Final clas
				Surgeon 1	Surgeon 2	Surgeon 3	Surgeon 1	Surgeon 2	Surgeon 3	Surgeon 1	Surgeon 2	Surgeon 3	Surgeon 1	Surgeon 2	Surgeon 3	Surgeon 1	Surgeon 2	Surgeon 3		Surgeon 1	Surgeon 2	Surgeon 3	
1	R5	W2	1.13	3	3	3	3	3	3	3	3	3	2	3	2	11	12	10	11	SUS	UN	SUS	SUS
2	R4	W2	1.04	1	3	2	2	2	2	2	3	2	1	1	1	6	9	7	7.3	NU	NU	NU	NU
3	R1	W3	1.03	2	2	2	2	2	2	3	3	3	2	1	2	9	8	9	8.7	NU	NU	NU	NU
4	R2	W3	1.20	2	2	2	3	2	3	3	3	3	1	1	1	9	8	9	8.7	NU	NU	NU	NU
5	R5	W3	1.28	3	3	2	3	3	3	3	3	3	3	2	2	12	11	10	11	UN	SUS	SUS	SUS
6	R4	W3	1.11	3	3	3	3	3	3	2	2	2	2	1	1	10	9	9	9.3	SUS	NU	NU	NU
7	R5	W4	1.34	3	3	3	3	3	3	3	3	3	1	1	1	10	10	10	10	SUS	SUS	SUS	SUS
8	R4	W4	1.11	3	3	2	3	3	3	3	4	2	3	4	3	12	14	10	12	UN	UN	SUS	UN
9	R3	W5	1.02	3	2	2	3	2	2	3	3	3	1	1	2	10	8	9	9	SUS	NU	NU	NU
10	R7	W5	1.28	3	3	3	3	3	3	3	3	3	1	1	1	10	10	10	10	SUS	SUS	SUS	SUS
11	R6	W5	1.20	3	3	2	3	3	2	3	3	3	3	3	2	12	12	9	11.7	UN	UN	UN	UN
12	R6	W5	1.58	3	1	2	3	1	3	3	3	3	3	1	3	12	6	11	9	UN	NU	SUS	NU
13	R5	W5	1.40	3	3	3	3	3	3	3	3	3	2	2	3	11	11	12	11.3	SUS	SUS	UN	SUS
14	R4	W5	1.09	3	2	2	3	3	2	3	4	3	3	4	3	12	13	10	11.7	UN	UN	SUS	UN
15	R3	W6	1.23	3	3	3	3	3	3	3	3	2	3	3	3	12	12	11	11.7	UN	UN	SUS	UN
16	R7	W6	1.46	3	1	2	3	2	3	3	3	3	1	1	2	10	7	10	9	SUS	NU	SUS	NU
17	R8	W6	1.24	3	2	2	3	3	3	3	3	3	3	3	3	12	11	11	11.3	UN	SUS	SUS	SUS
18	R6	W6	1.63	3	2	3	3	3	3	3	3	3	3	3	3	12	11	12	11.7	UN	SUS	UN	UN
19	R7	W7	1.50	3	2	2	3	3	3	3	3	3	1	1	2	10	9	10	9.7	SUS	NU	SUS	NU
20	R8	W7	1.28	2	2	3	3	3	3	3	3	3	3	3	3	11	11	12	11.3	SUS	SUS	UN	SUS
21	R6	W7	1.56	3	2	3	3	3	3	3	3	3	3	3	3	12	11	12	11.7	UN	SUS	UN	UN
22	R7	W8	1.38	3	3	3	3	3	3	3	3	4	1	1	2	10	10	12	10.7	SUS	SUS	UN	SUS
23	R8	W8	1.44	1	4	2	3	3	3	3	3	4	3	3	4	10	13	13	12	SUS	UN	UN	UN
24	R7	W9	1.43	3	3	3	3	3	3	3	3	3	1	4	4	10	13	13	12	SUS	UN	UN	UN
25	R8	W9	1.74	3	4	4	3	3	3	3	3	3	3	3	3	12	13	13	12.7	UN	UN	UN	UN

## Data Availability

The data are available at the Orthopedics Surgeons Association or Orthopedics Academy.

## Supplementary Materials

Supplementary Materials Table A: the RUST form, which included 25 pairs of radiographs (oblique and anterior-posterior views) of eight rabbits.
