# Supplementary material for: Bone Healing Monitoring in Bone Lengthening Using Bioimpedance
Source: J Healthc Eng. 2022 Apr 7;2022:3226440. doi: 10.1155/2022/3226440 (PMC9010194; doi:10.1155/2022/3226440)
Supplement: Supplementary Materials — Table A: the RUST form, which included 25 pairs of radiographs (oblique and anterior-posterior views) of eight rabbits. [file 3226440.f1.pdf]

Table A. the RUST form, which included 25 pairs of radiographs (oblique and anterior-posterior views) of eight rabbits

	Oblique View					Anterior-Posterior View				
	cortex	no callus	Callus present	bridging callus	remodeled, fracture not visible	cortex	no callus	Callus present	bridging callus	remodeled, fracture not visible
	**Right cortex**	Right cortex score	Right cortex score	Right cortex score	Right cortex score	**Right cortex**	Right cortex score	Right cortex score	Right cortex score	Right cortex score
	**Left cortex**	Left cortex score	Left cortex score	Left cortex score	Left cortex score	**Left cortex**	Left cortex score	Left cortex score	Left cortex score	Left cortex score
Rabbit 5<br>Week 2	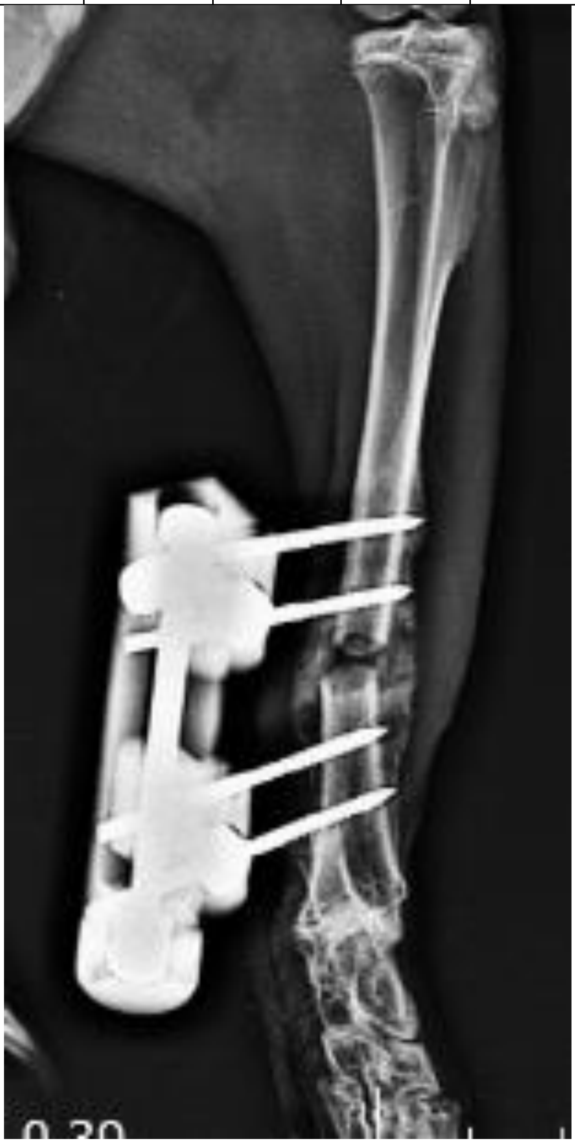					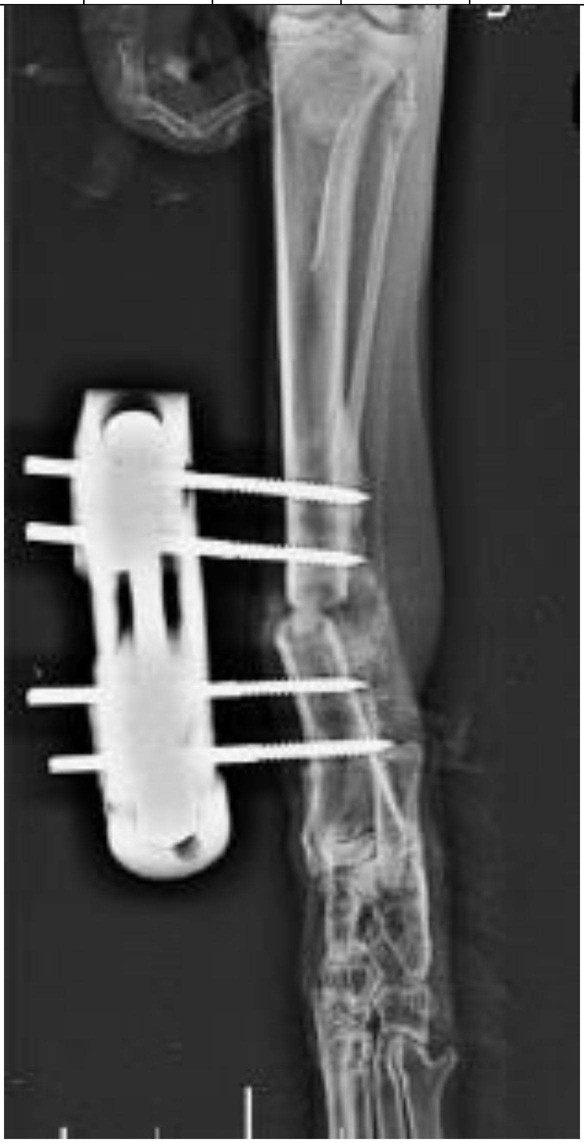				

	Oblique View					Anterior-Posterior View				
	cortex	no callus	Callus present	bridging callus	remodeled, fracture not visible	cortex	no callus	Callus present	bridging callus	remodeled, fracture not visible
	**Right cortex**	Right cortex score	Right cortex score	Right cortex score	Right cortex score	**Right cortex**	Right cortex score	Right cortex score	Right cortex score	Right cortex score
	**Left cortex**	Left cortex score	Left cortex score	Left cortex score	Left cortex score	**Left cortex**	Left cortex score	Left cortex score	Left cortex score	Left cortex score
Rabbit 4 Week 2	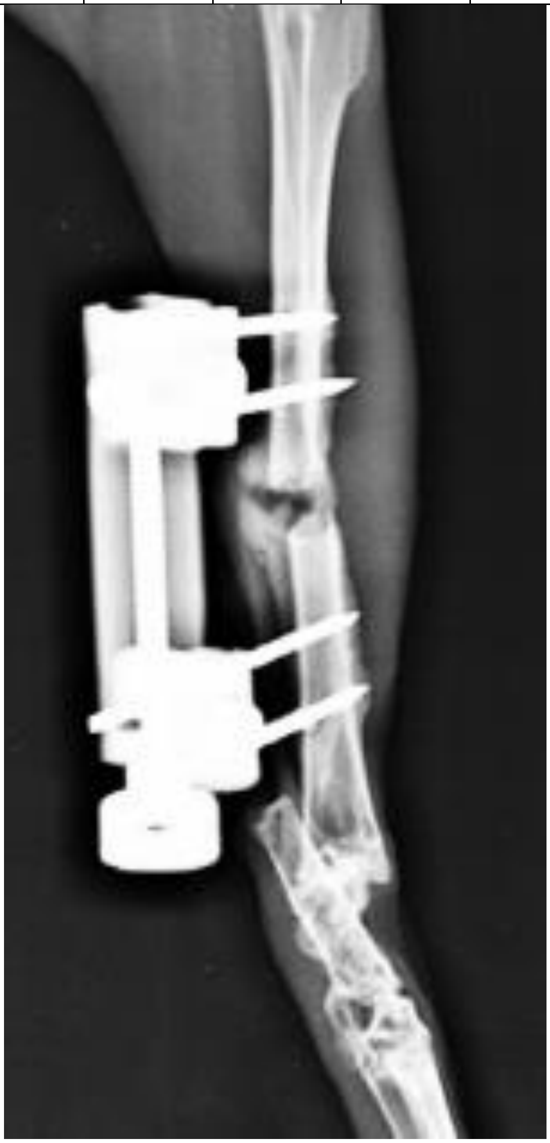					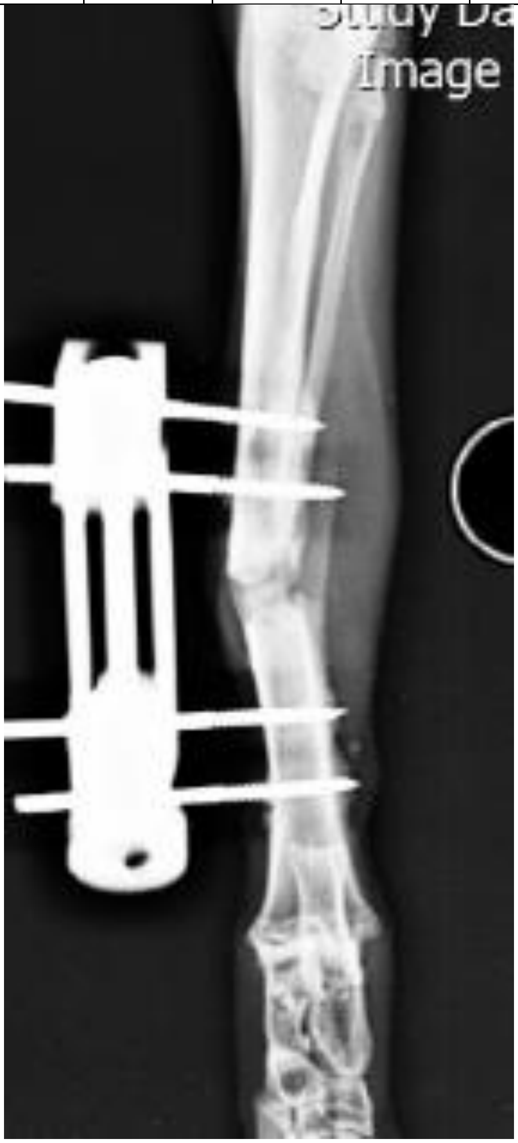				

	Oblique View					Anterior-Posterior View				
	cortex	no callus	Callus present	bridging callus	remodeled, fracture not visible	cortex	no callus	Callus present	bridging callus	remodeled, fracture not visible
	**Right cortex**	Right cortex score	Right cortex score	Right cortex score	Right cortex score	**Right cortex**	Right cortex score	Right cortex score	Right cortex score	Right cortex score
	**Left cortex**	Left cortex score	Left cortex score	Left cortex score	Left cortex score	**Left cortex**	Left cortex score	Left cortex score	Left cortex score	Left cortex score
Rabbit 1<br>Week 3	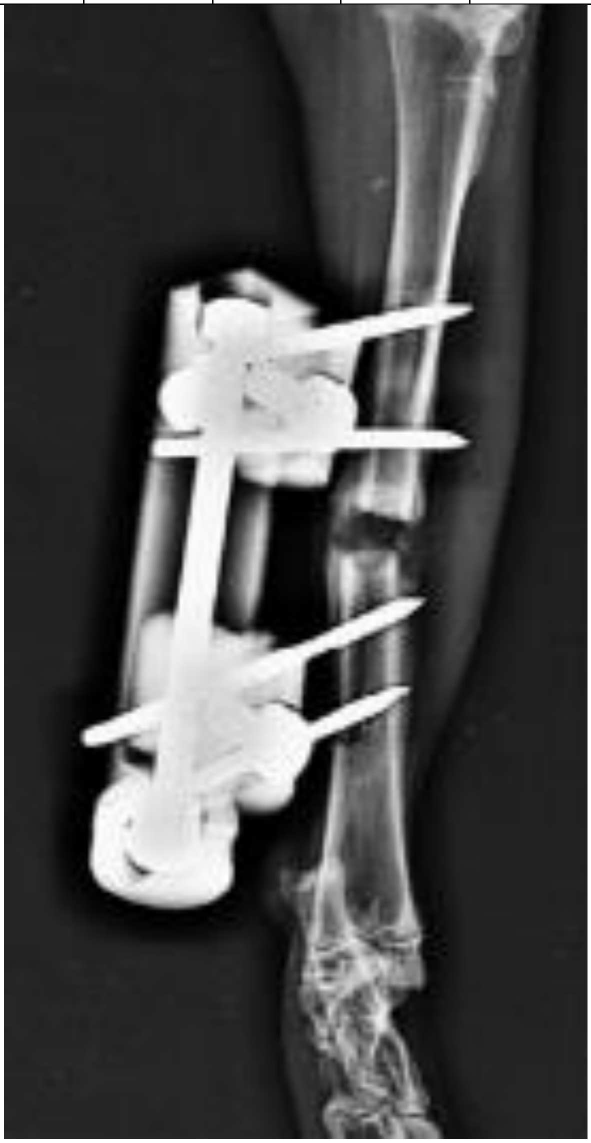					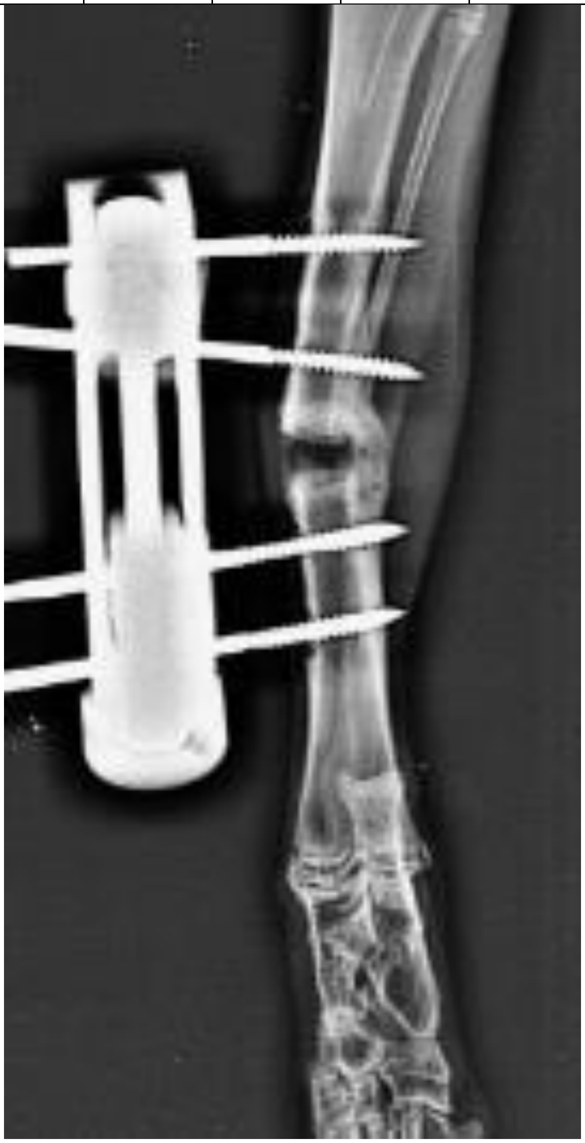				

Rabbit 2<br>Week 3	Oblique View					Anterior-Posterior View				
	cortex	no callus	Callus present	bridging callus	remodeled, fracture not visible	cortex	no callus	Callus present	bridging callus	remodeled, fracture not visible
	**Right cortex**	Right cortex score	Right cortex score	Right cortex score	Right cortex score	**Right cortex**	Right cortex score	Right cortex score	Right cortex score	Right cortex score
	**Left cortex**	Left cortex score	Left cortex score	Left cortex score	Left cortex score	**Left cortex**	Left cortex score	Left cortex score	Left cortex score	Left cortex score
	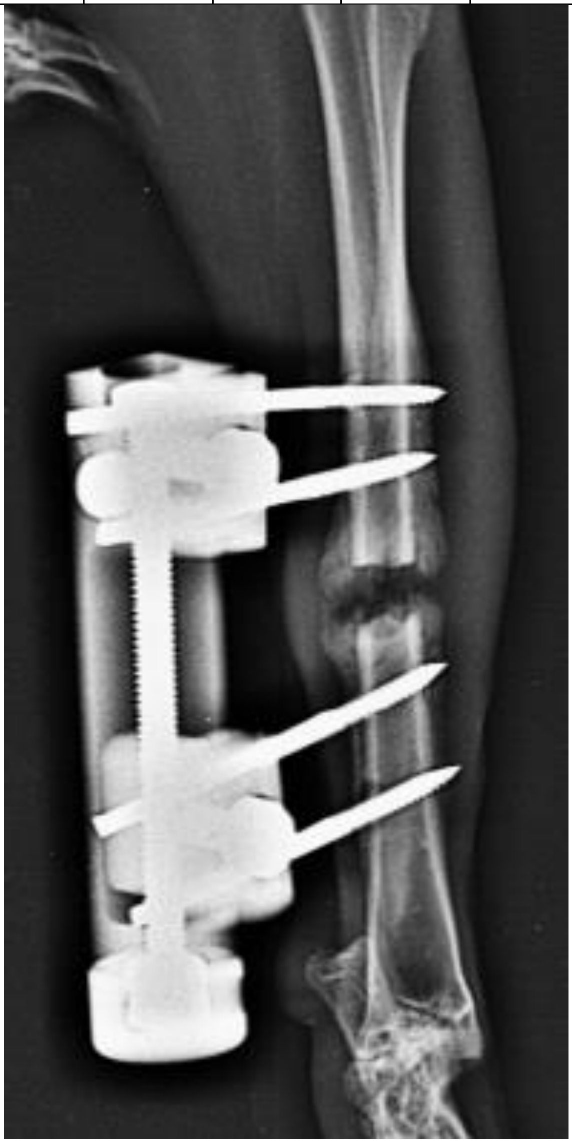					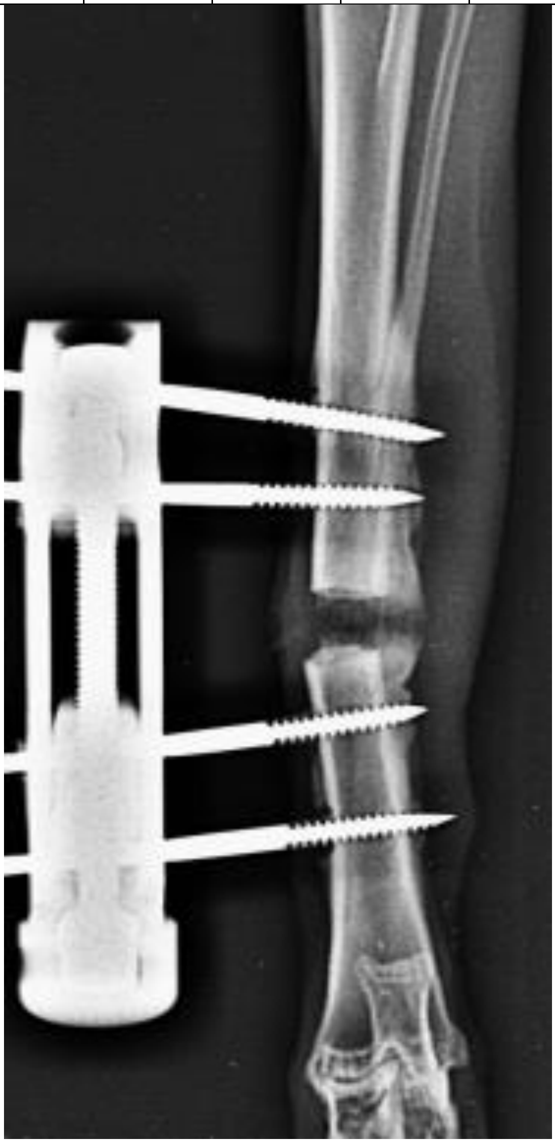				

	Oblique View					Anterior-Posterior View				
	cortex	no callus	Callus present	bridging callus	remodeled, fracture not visible	cortex	no callus	Callus present	bridging callus	remodeled, fracture not visible
	**Right cortex**	Right cortex score	Right cortex score	Right cortex score	Right cortex score	**Right cortex**	Right cortex score	Right cortex score	Right cortex score	Right cortex score
	**Left cortex**	Left cortex score	Left cortex score	Left cortex score	Left cortex score	**Left cortex**	Left cortex score	Left cortex score	Left cortex score	Left cortex score
Rabbit 5 Week 3	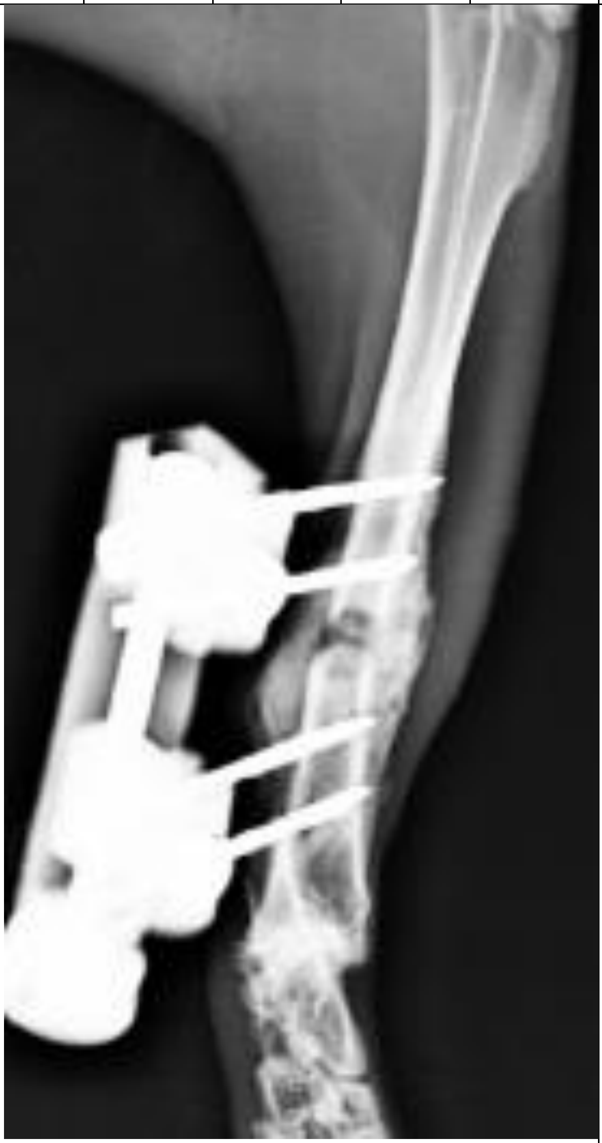					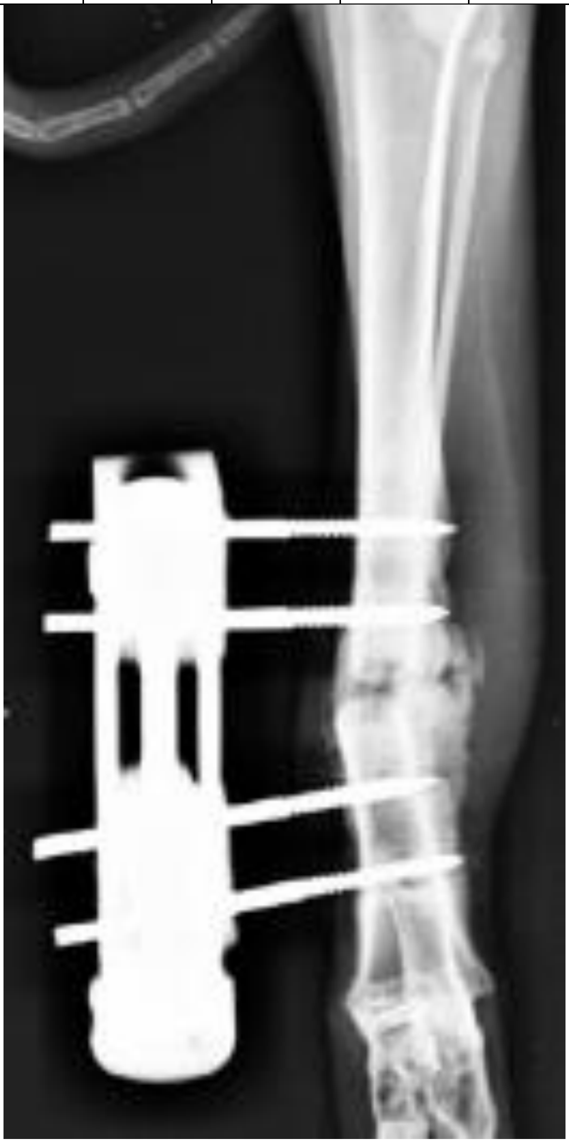				

	Oblique View					Anterior-Posterior View				
	cortex	no callus	Callus present	bridging callus	remodeled, fracture not visible	cortex	no callus	Callus present	bridging callus	remodeled, fracture not visible
	**Right cortex**	Right cortex score	Right cortex score	Right cortex score	Right cortex score	**Right cortex**	Right cortex score	Right cortex score	Right cortex score	Right cortex score
	**Left cortex**	Left cortex score	Left cortex score	Left cortex score	Left cortex score	**Left cortex**	Left cortex score	Left cortex score	Left cortex score	Left cortex score
Rabbit 4<br>Week 3	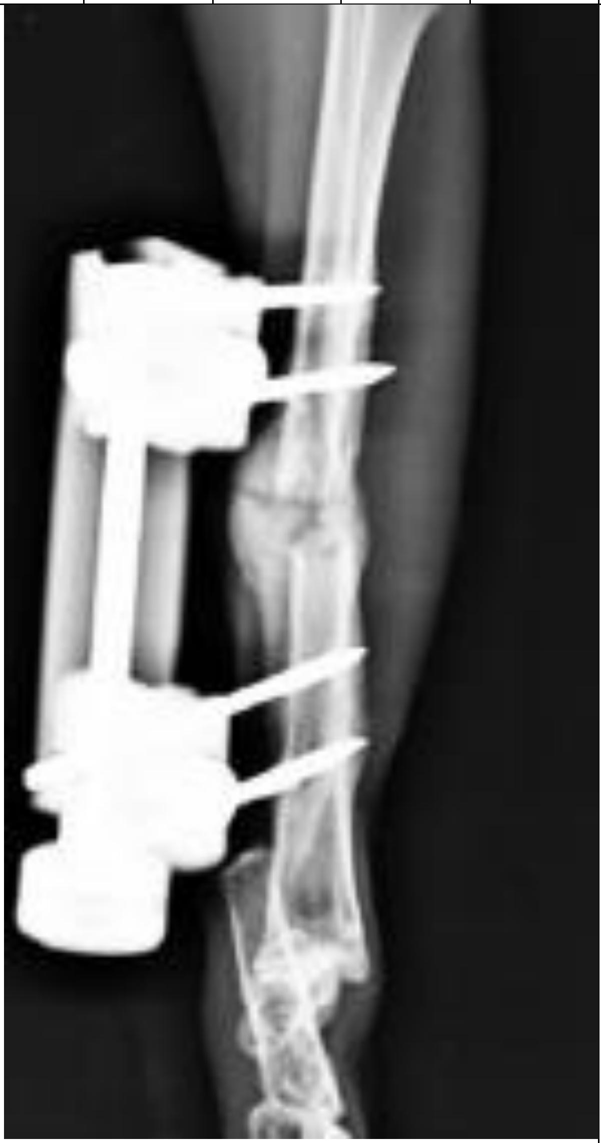					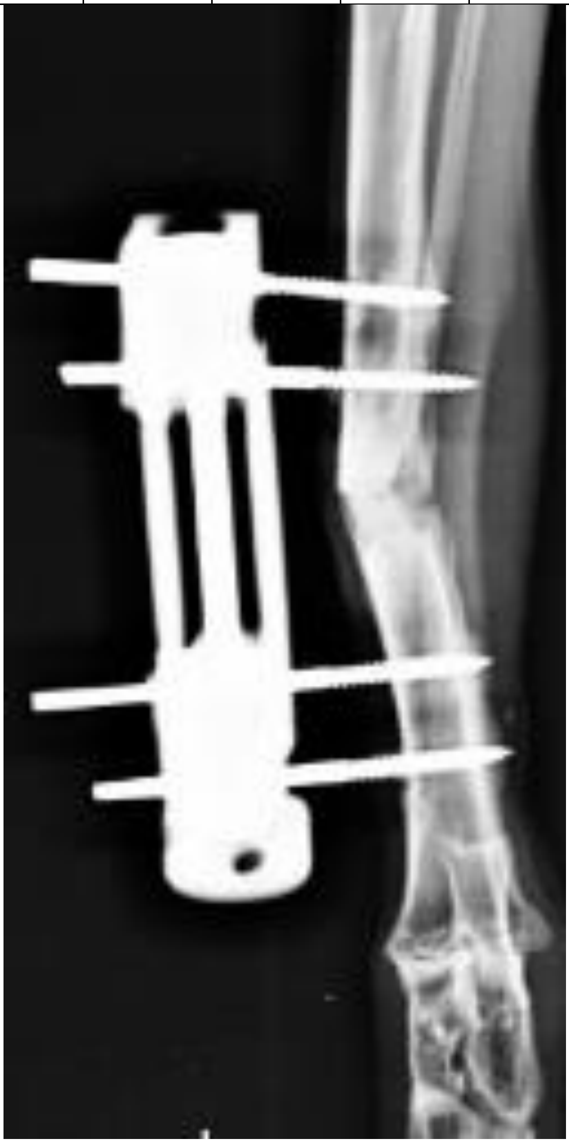				

<table>
<tr><td rowspan="5">Rabbit 5
Week 4</td><td colspan="5">Oblique View</td><td colspan="5">Anterior-Posterior View</td></tr>
<tr><td>cortex</td><td>no callus</td><td>Callus present</td><td>bridging callus</td><td>remodeled, fracture not visible</td><td>cortex</td><td>no callus</td><td>Callus present</td><td>bridging callus</td><td>remodeled, fracture not visible</td></tr>
<tr><td>Right cortex</td><td>Right cortex score</td><td>Right cortex score</td><td>Right cortex score</td><td>Right cortex score</td><td>Right cortex</td><td>Right cortex score</td><td>Right cortex score</td><td>Right cortex score</td><td>Right cortex score</td></tr>
<tr><td>Left cortex</td><td>Left cortex score</td><td>Left cortex score</td><td>Left cortex score</td><td>Left cortex score</td><td>Left cortex</td><td>Left cortex score</td><td>Left cortex score</td><td>Left cortex score</td><td>Left cortex score</td></tr>
<tr><td colspan="5">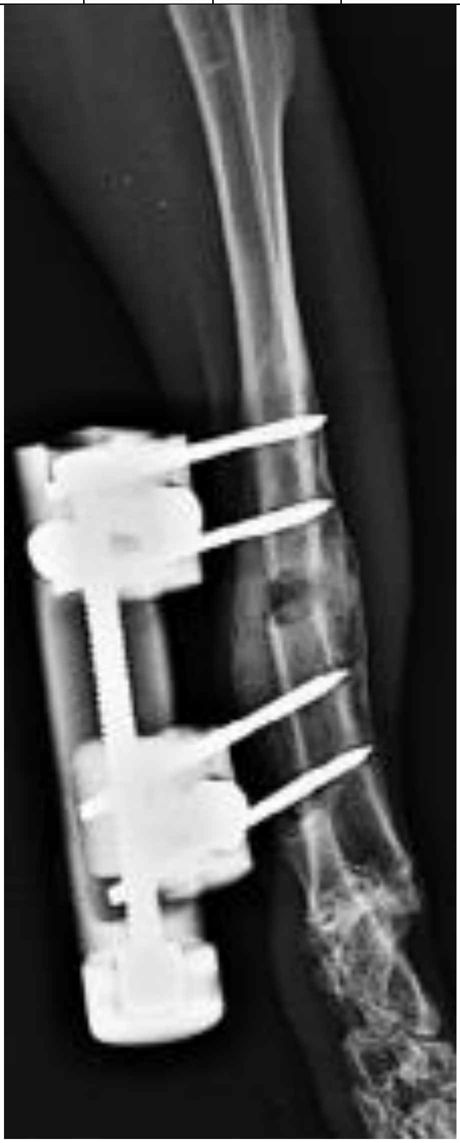</td><td colspan="5">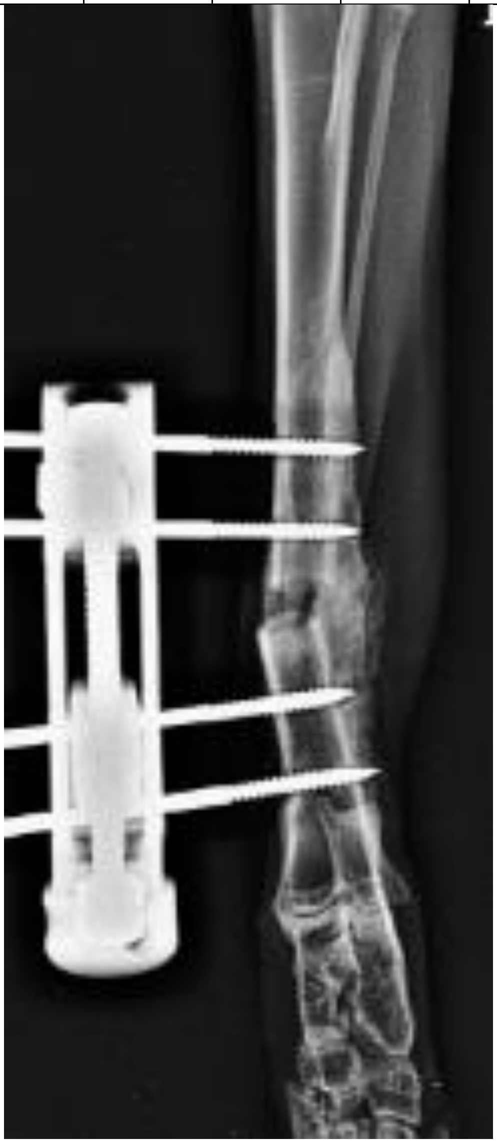</td></tr>
</table>

	Oblique View					Anterior-Posterior View				
	cortex	no callus	Callus present	bridging callus	remodeled, fracture not visible	cortex	no callus	Callus present	bridging callus	remodeled, fracture not visible
	**Right cortex**	Right cortex score	Right cortex score	Right cortex score	Right cortex score	**Right cortex**	Right cortex score	Right cortex score	Right cortex score	Right cortex score
	**Left cortex**	Left cortex score	Left cortex score	Left cortex score	Left cortex score	**Left cortex**	Left cortex score	Left cortex score	Left cortex score	Left cortex score
Rabbit 4 Week 4	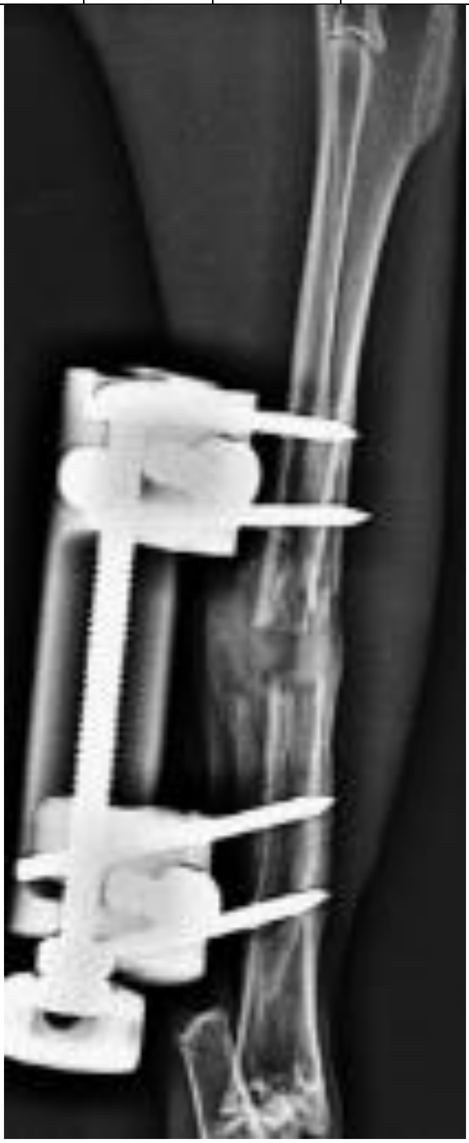					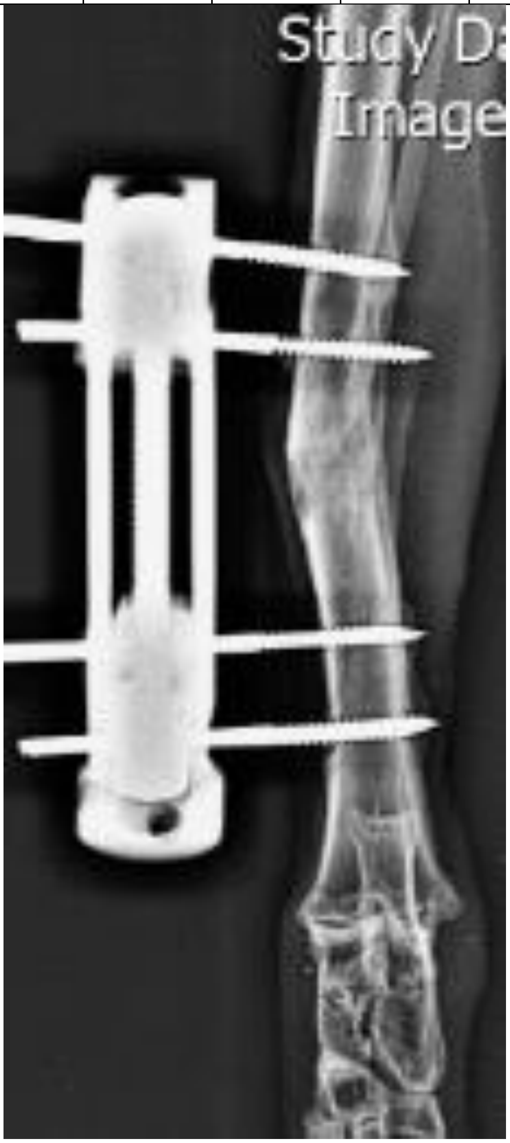				

	Oblique View					Anterior-Posterior View				
	cortex	no callus	Callus present	bridging callus	remodeled, fracture not visible	cortex	no callus	Callus present	bridging callus	remodeled, fracture not visible
	**Right cortex**	Right cortex score	Right cortex score	Right cortex score	Right cortex score	**Right cortex**	Right cortex score	Right cortex score	Right cortex score	Right cortex score
	**Left cortex**	Left cortex score	Left cortex score	Left cortex score	Left cortex score	**Left cortex**	Left cortex score	Left cortex score	Left cortex score	Left cortex score
Rabbit 3<br>Week 5	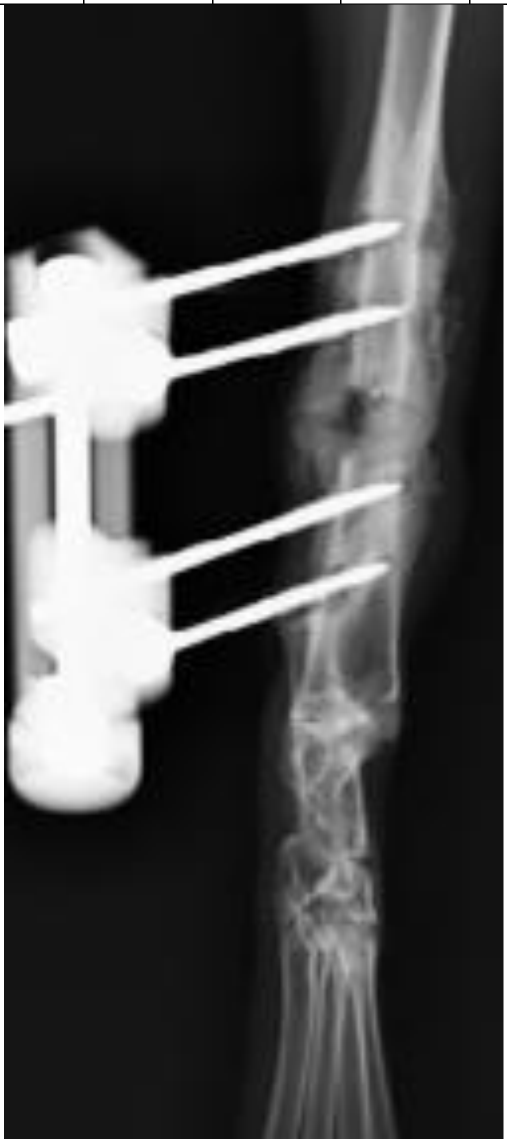					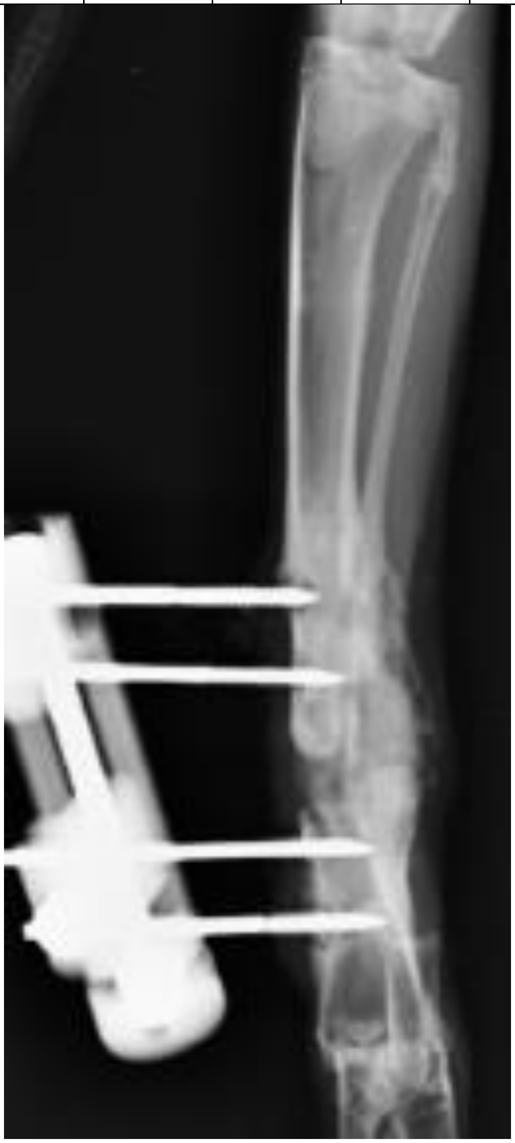				

	Oblique View					Anterior-Posterior View				
	cortex	no callus	Callus present	bridging callus	remodeled, fracture not visible	cortex	no callus	Callus present	bridging callus	remodeled, fracture not visible
	**Right cortex**	Right cortex score	Right cortex score	Right cortex score	Right cortex score	**Right cortex**	Right cortex score	Right cortex score	Right cortex score	Right cortex score
	**Left cortex**	Left cortex score	Left cortex score	Left cortex score	Left cortex score	**Left cortex**	Left cortex score	Left cortex score	Left cortex score	Left cortex score
Rabbit 7<br>Week 5	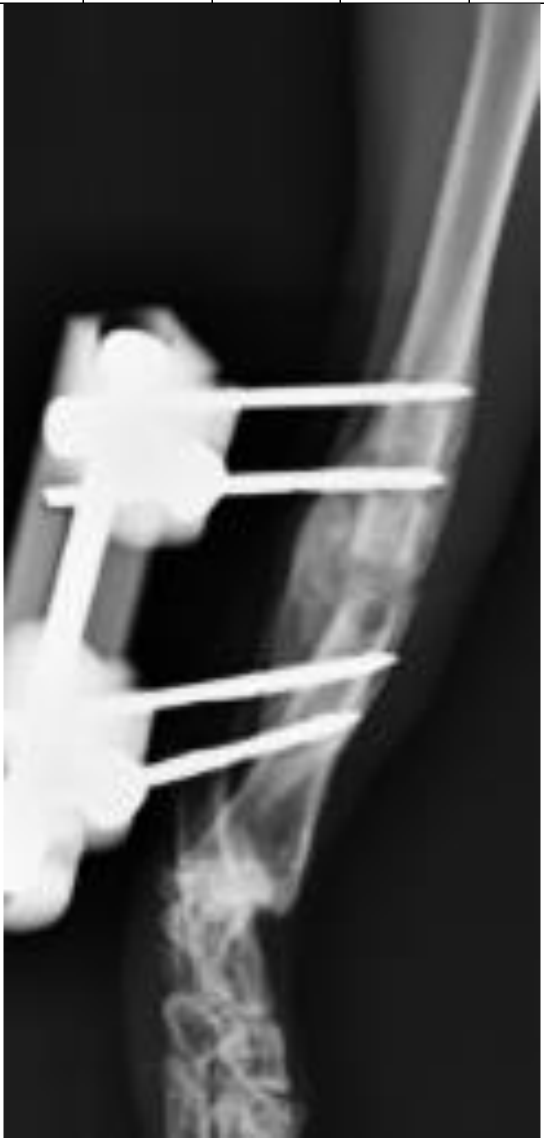					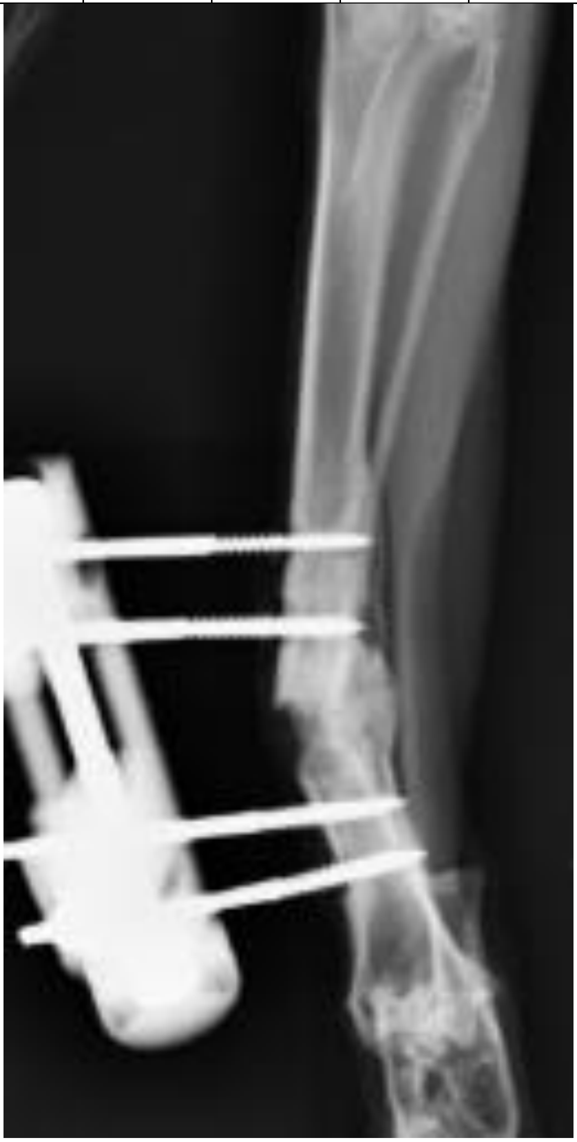				

	Oblique View					Anterior-Posterior View				
	cortex	no callus	Callus present	bridging callus	remodeled, fracture not visible	cortex	no callus	Callus present	bridging callus	remodeled, fracture not visible
	**Right cortex**	Right cortex score	Right cortex score	Right cortex score	Right cortex score	**Right cortex**	Right cortex score	Right cortex score	Right cortex score	Right cortex score
	**Left cortex**	Left cortex score	Left cortex score	Left cortex score	Left cortex score	**Left cortex**	Left cortex score	Left cortex score	Left cortex score	Left cortex score
Rabbit 3<br>Week 5	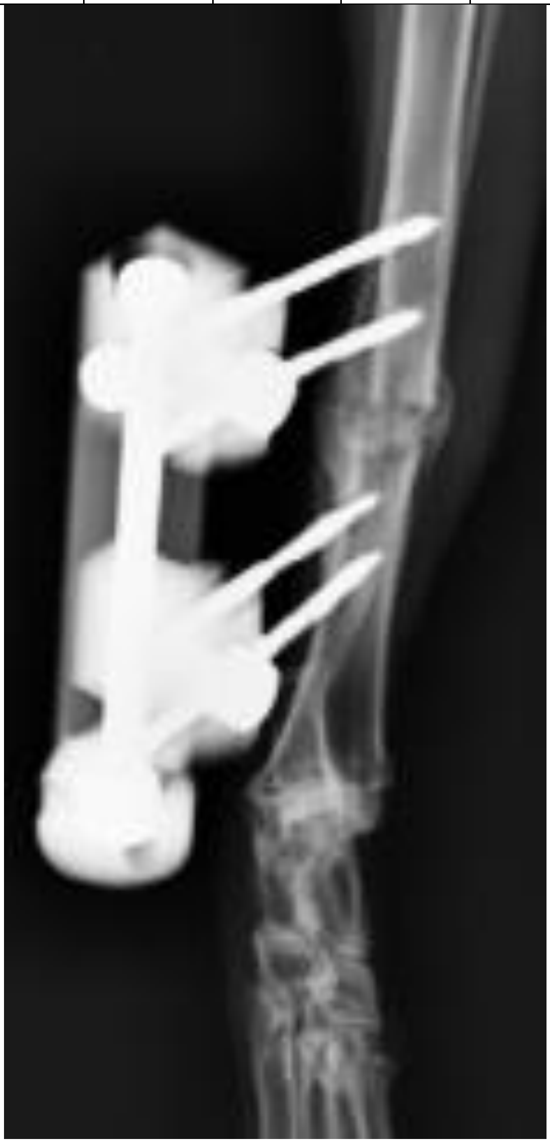					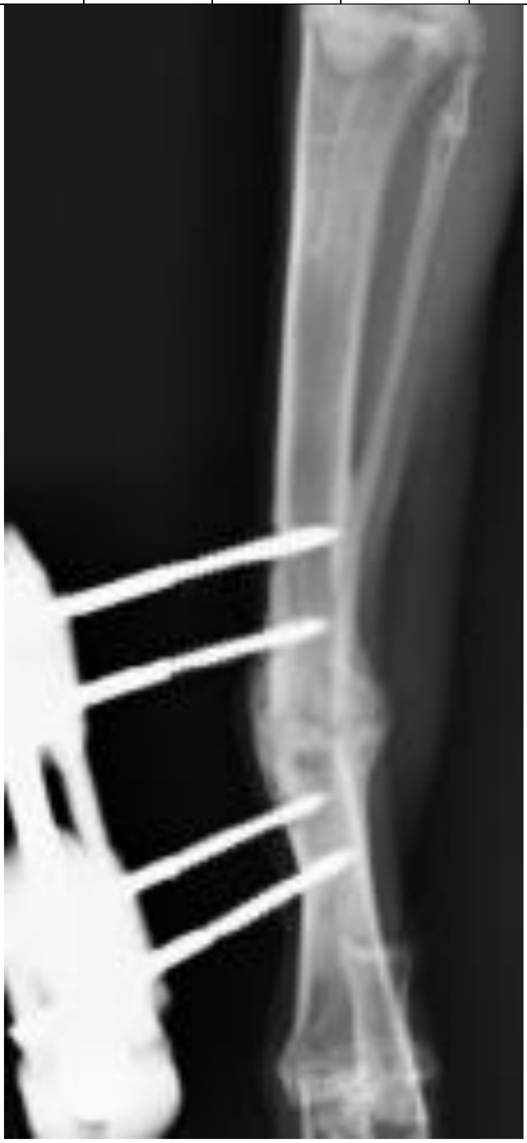				

	Oblique View					Anterior-Posterior View				
	cortex	no callus	Callus present	bridging callus	remodeled, fracture not visible	cortex	no callus	Callus present	bridging callus	remodeled, fracture not visible
	**Right cortex**	Right cortex score	Right cortex score	Right cortex score	Right cortex score	**Right cortex**	Right cortex score	Right cortex score	Right cortex score	Right cortex score
	**Left cortex**	Left cortex score	Left cortex score	Left cortex score	Left cortex score	**Left cortex**	Left cortex score	Left cortex score	Left cortex score	Left cortex score
Rabbir 6<br>Week 5	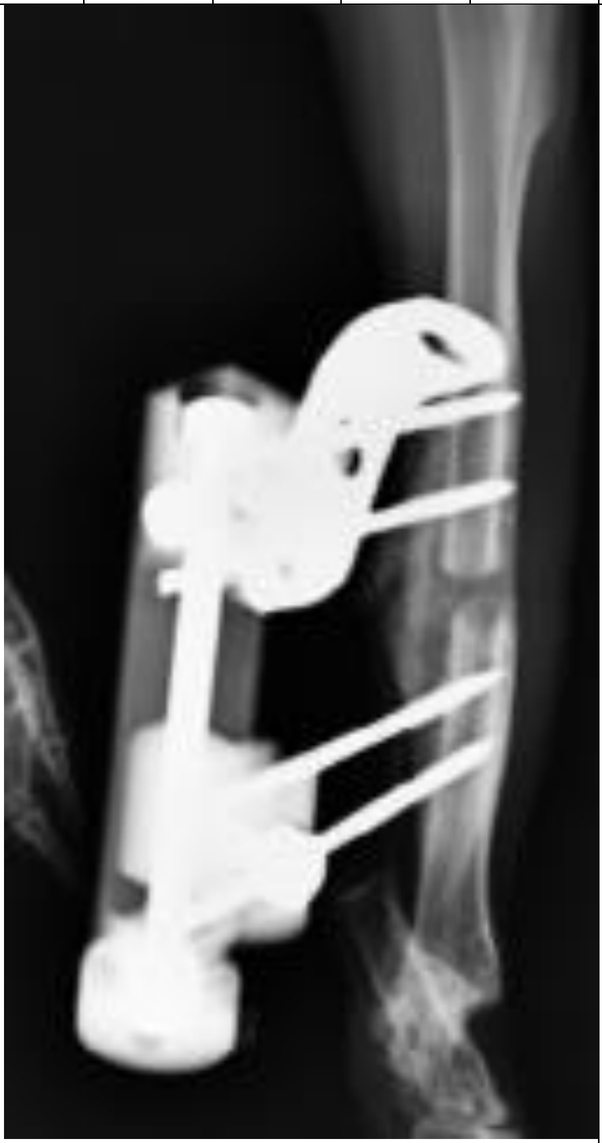					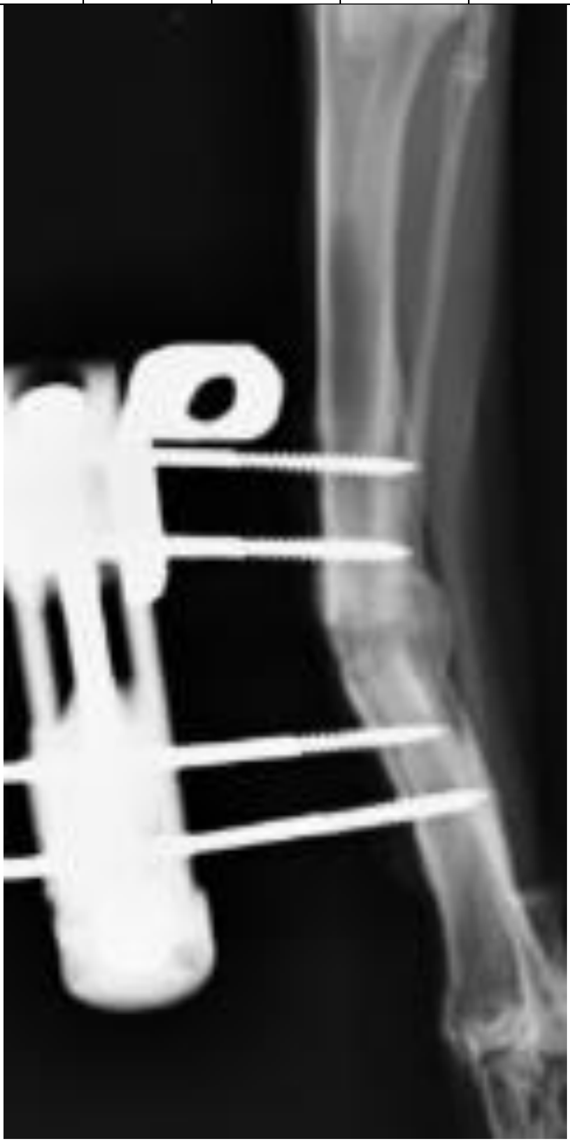				

	Oblique View					Anterior-Posterior View				
	cortex	no callus	Callus present	bridging callus	remodeled, fracture not visible	cortex	no callus	Callus present	bridging callus	remodeled, fracture not visible
	**Right cortex**	Right cortex score	Right cortex score	Right cortex score	Right cortex score	**Right cortex**	Right cortex score	Right cortex score	Right cortex score	Right cortex score
	**Left cortex**	Left cortex score	Left cortex score	Left cortex score	Left cortex score	**Left cortex**	Left cortex score	Left cortex score	Left cortex score	Left cortex score
Rabbit 5<br>Week 5	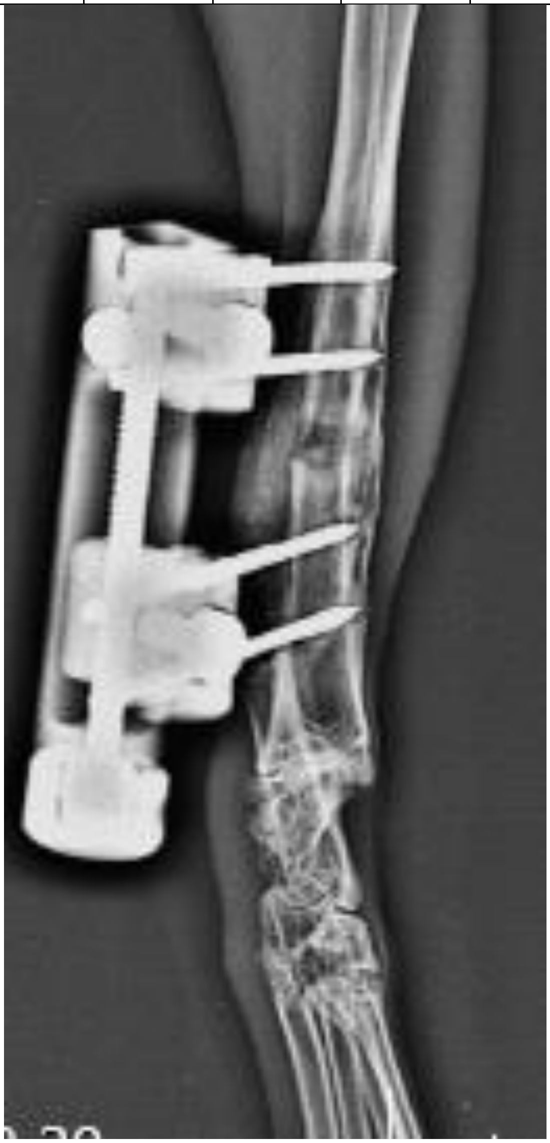					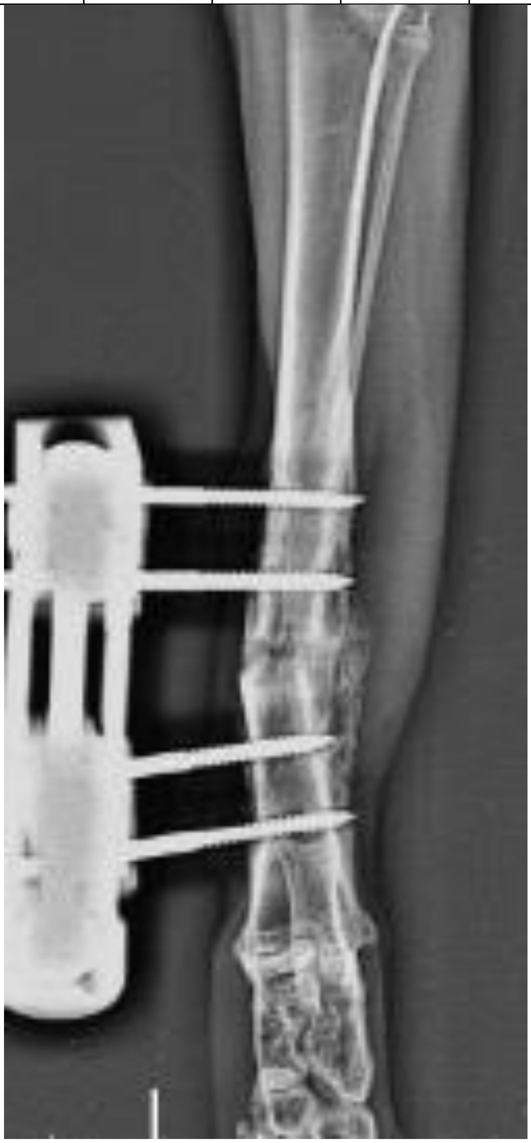				

Rabbit 4<br><br>Week 5	Oblique View					Anterior-Posterior View				
	cortex	no callus	Callus present	bridging callus	remodeled, fracture not visible	cortex	no callus	Callus present	bridging callus	remodeled, fracture not visible
	**Right cortex**	Right cortex score	Right cortex score	Right cortex score	Right cortex score	**Right cortex**	Right cortex score	Right cortex score	Right cortex score	Right cortex score
	**Left cortex**	Left cortex score	Left cortex score	Left cortex score	Left cortex score	**Left cortex**	Left cortex score	Left cortex score	Left cortex score	Left cortex score
	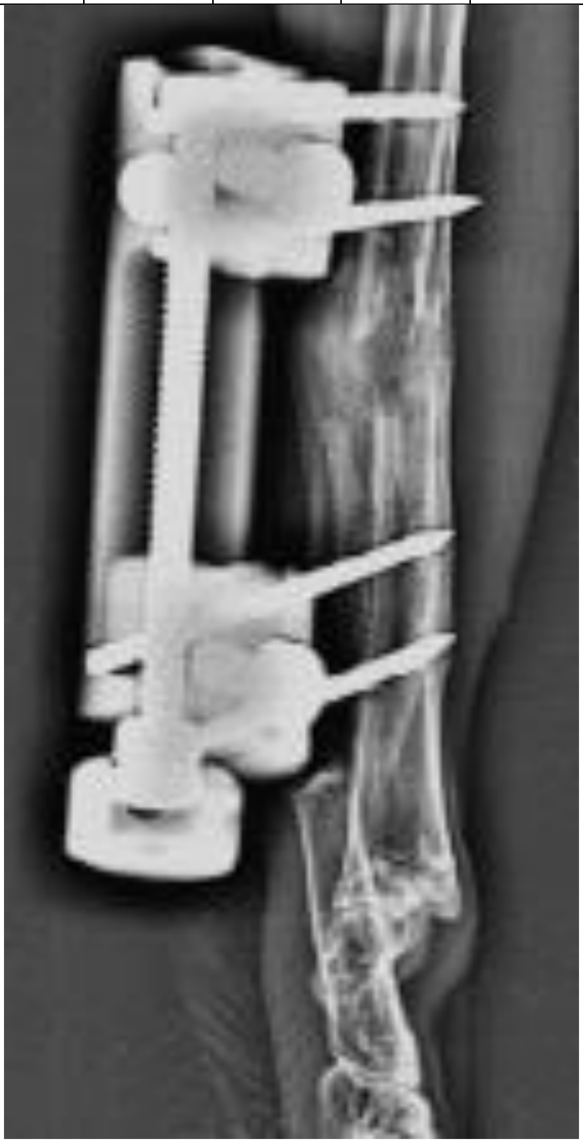					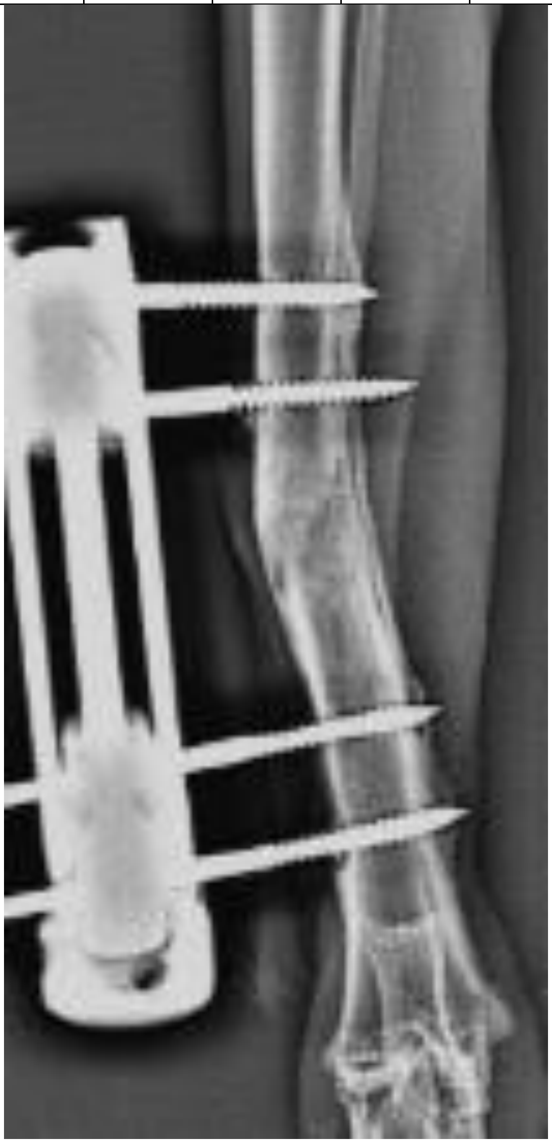				

	Oblique View					Anterior-Posterior View				
	cortex	no callus	Callus present	bridging callus	remodeled, fracture not visible	cortex	no callus	Callus present	bridging callus	remodeled, fracture not visible
	**Right cortex**	Right cortex score	Right cortex score	Right cortex score	Right cortex score	**Right cortex**	Right cortex score	Right cortex score	Right cortex score	Right cortex score
	**Left cortex**	Left cortex score	Left cortex score	Left cortex score	Left cortex score	**Left cortex**	Left cortex score	Left cortex score	Left cortex score	Left cortex score
Rabbit 3<br>Week 6	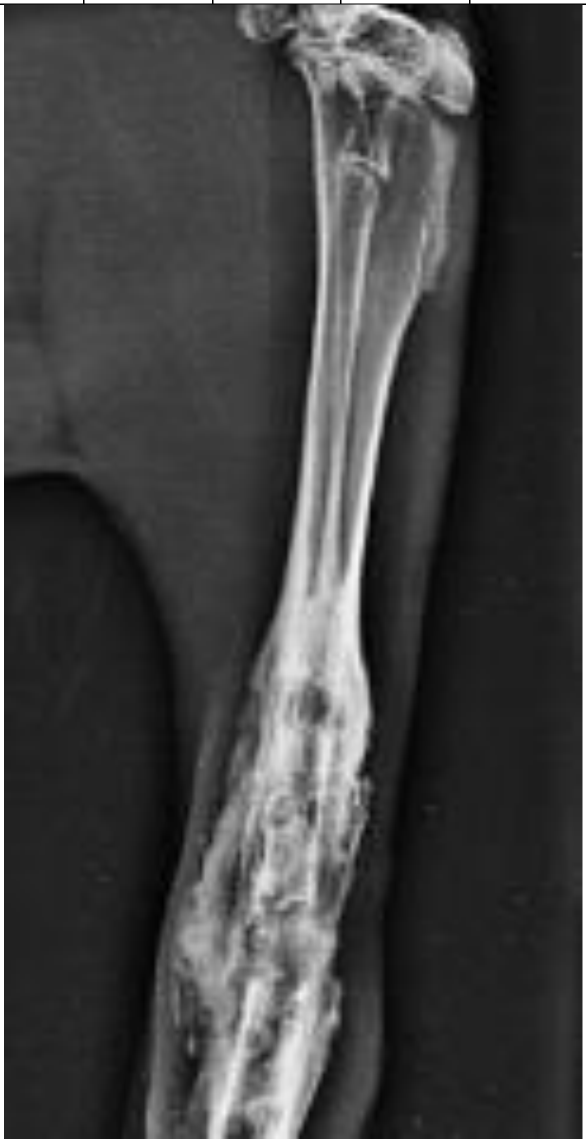					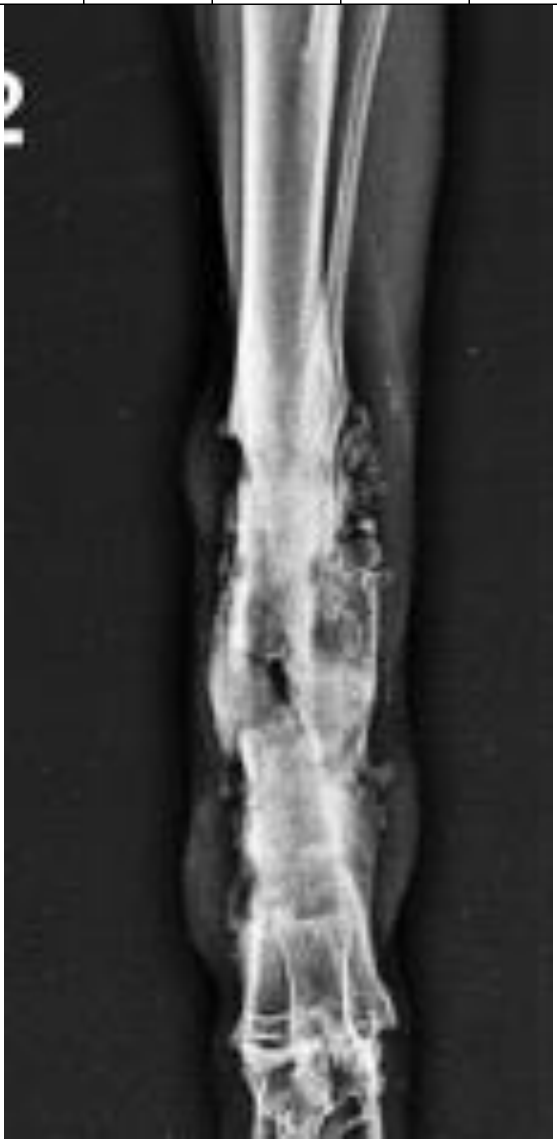				

	Oblique View					Anterior-Posterior View				
	cortex	no callus	Callus present	bridging callus	remodeled, fracture not visible	cortex	no callus	Callus present	bridging callus	remodeled, fracture not visible
	**Right cortex**	Right cortex score	Right cortex score	Right cortex score	Right cortex score	**Right cortex**	Right cortex score	Right cortex score	Right cortex score	Right cortex score
	**Left cortex**	Left cortex score	Left cortex score	Left cortex score	Left cortex score	**Left cortex**	Left cortex score	Left cortex score	Left cortex score	Left cortex score
Rabbit 7<br>Week 6	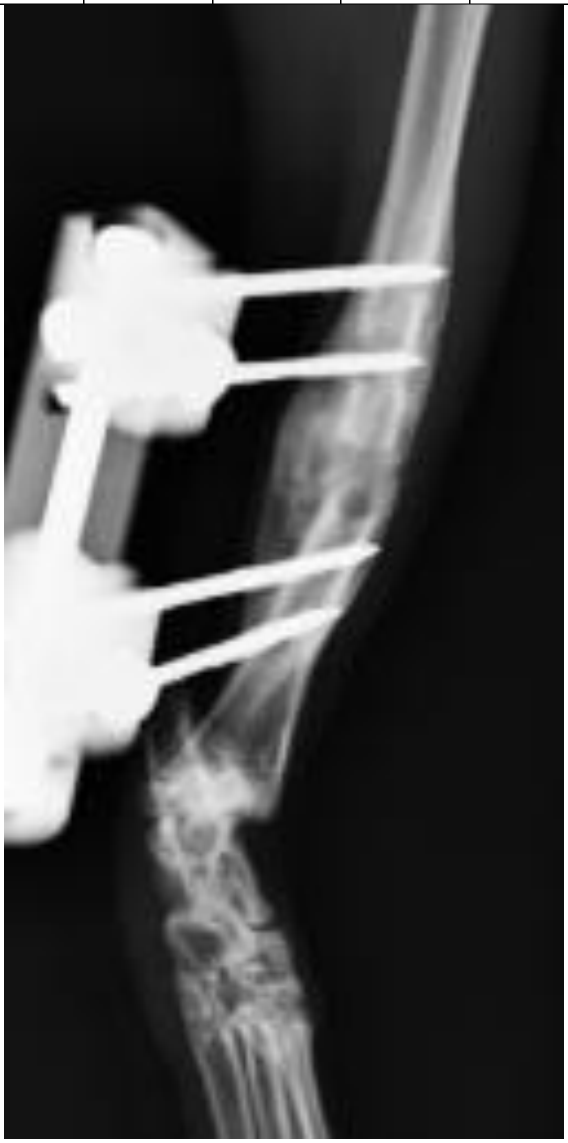					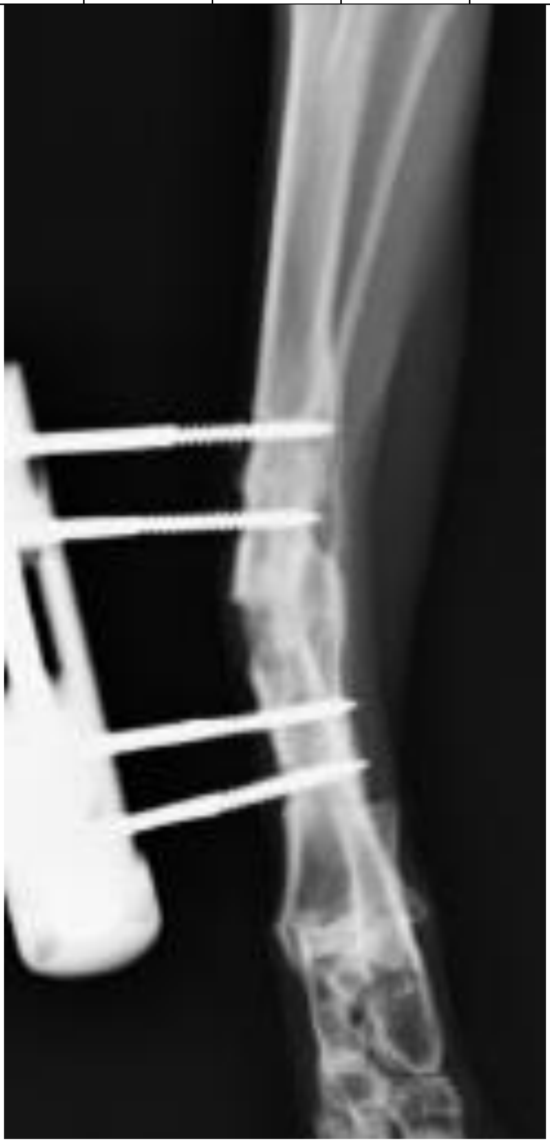				

	Oblique View					Anterior-Posterior View				
	cortex	no callus	Callus present	bridging callus	remodeled, fracture not visible	cortex	no callus	Callus present	bridging callus	remodeled, fracture not visible
	**Right cortex**	Right cortex score	Right cortex score	Right cortex score	Right cortex score	**Right cortex**	Right cortex score	R4	Right cortex score	Right cortex score
	**Left cortex**	Left cortex score	Left cortex score	Left cortex score	Left cortex score	**Left cortex**	Left cortex score	R3	Left cortex score	Left cortex score
Rabbit 8<br>Week 6	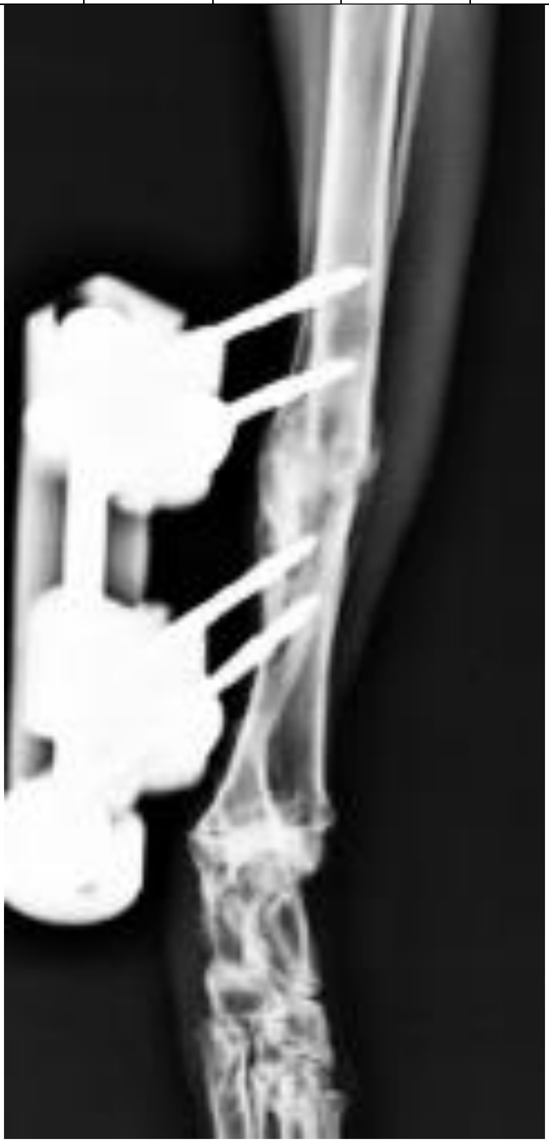					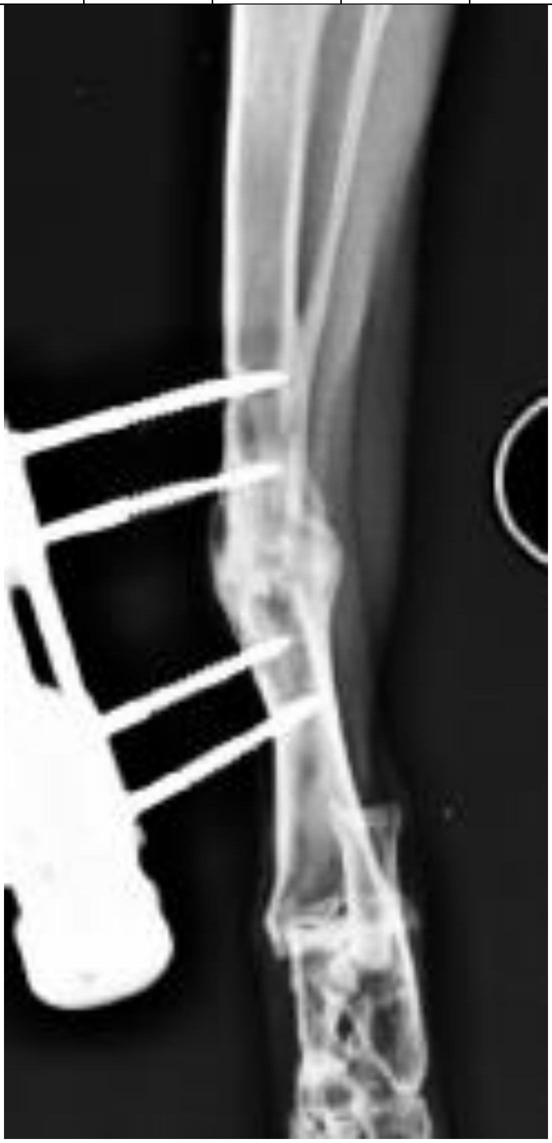				

	Oblique View					Anterior-Posterior View				
	cortex	no callus	Callus present	bridging callus	remodeled, fracture not visible	cortex	no callus	Callus present	bridging callus	remodeled, fracture not visible
	**Right cortex**	Right cortex score	Right cortex score	Right cortex score	Right cortex score	**Right cortex**	Right cortex score	R5	Right cortex score	Right cortex score
	**Left cortex**	Left cortex score	Left cortex score	Left cortex score	Left cortex score	**Left cortex**	Left cortex score	R4	Left cortex score	Left cortex score
Rabbit 6<br>Week 6	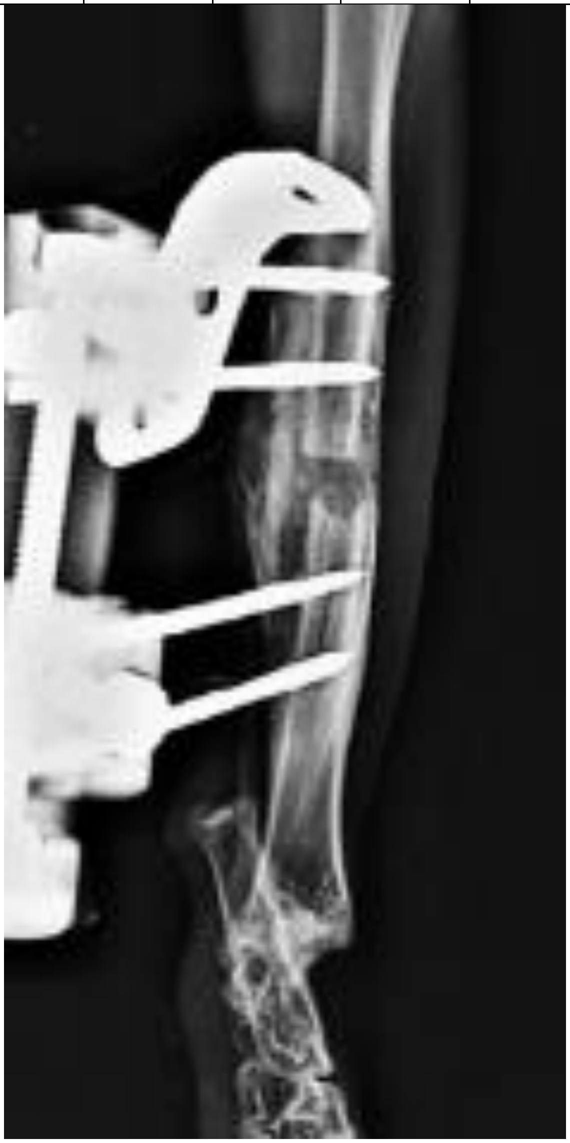					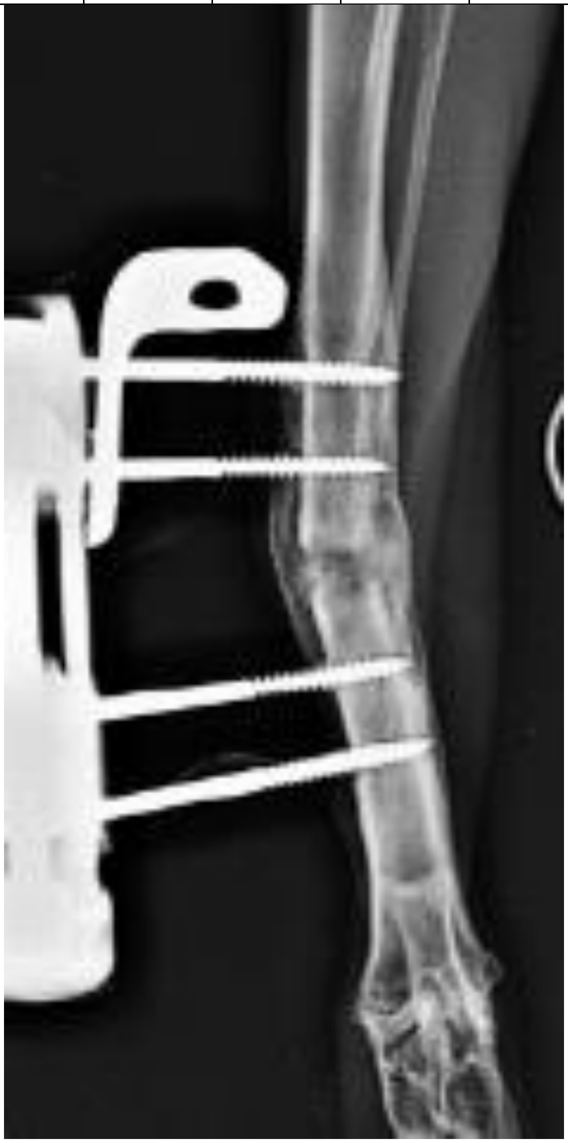				

	Oblique View					Anterior-Posterior View				
	cortex	no callus	Callus present	bridging callus	remodeled, fracture not visible	cortex	no callus	Callus present	bridging callus	remodeled, fracture not visible
	**Right cortex**	Right cortex score	Right cortex score	Right cortex score	Right cortex score	**Right cortex**	Right cortex score	RABBIT 6	Right cortex score	Right cortex score
	**Left cortex**	Left cortex score	Left cortex score	Left cortex score	Left cortex score	**Left cortex**	Left cortex score	R7	Left cortex score	Left cortex score
Rabbit 7<br>Week 7	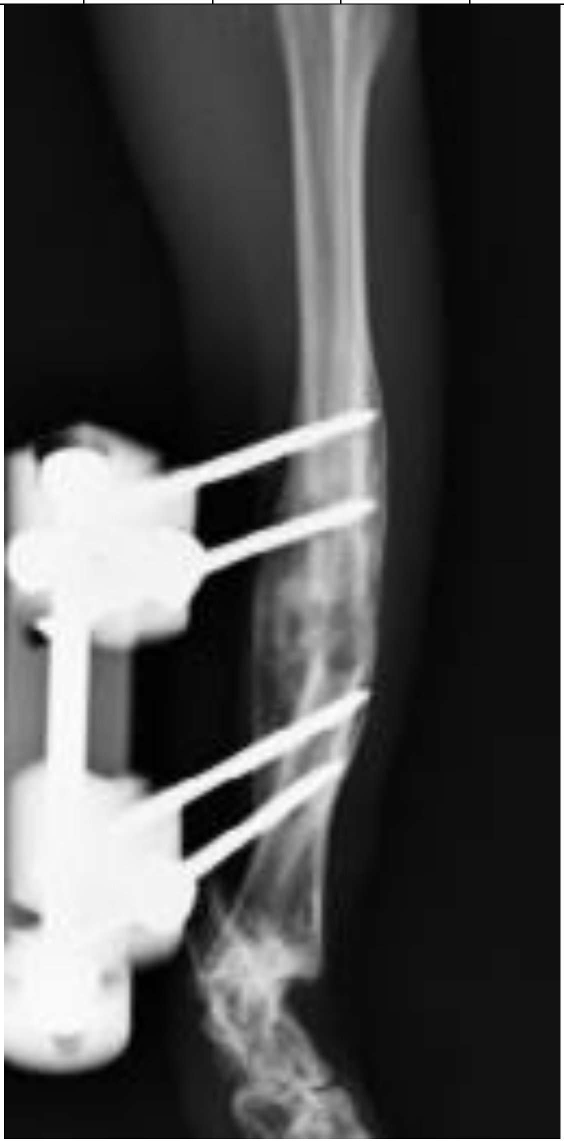					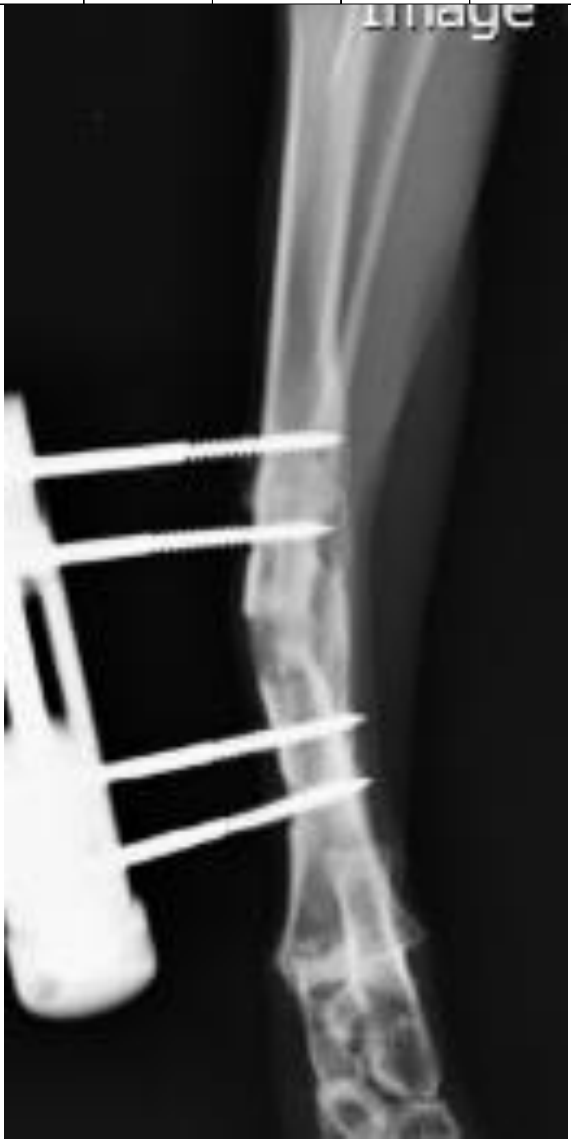				

	Oblique View					Anterior-Posterior View				
	cortex	no callus	Callus present	bridging callus	remodeled, fracture not visible	cortex	no callus	Callus present	bridging callus	remodeled, fracture not visible
	**Right cortex**	Right cortex score	Right cortex score	Right cortex score	Right cortex score	**Right cortex**	Right cortex score	R8	Right cortex score	Right cortex score
	**Left cortex**	Left cortex score	Left cortex score	Left cortex score	Left cortex score	**Left cortex**	Left cortex score	R7	Left cortex score	Left cortex score
Rabbit 8 Week 7	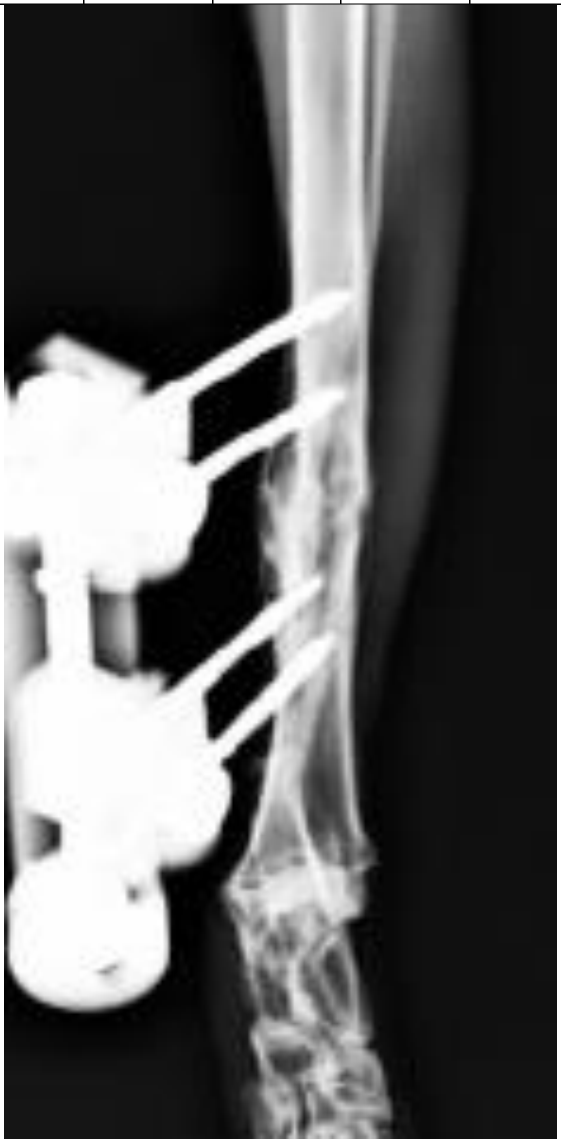					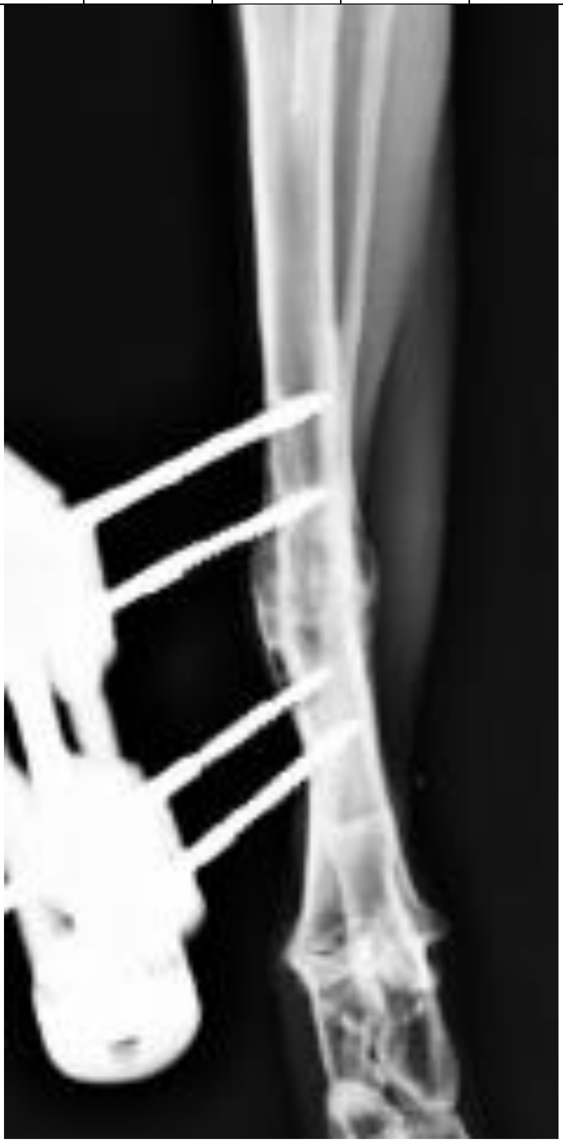				

	Oblique View					Anterior-Posterior View				
	cortex	no callus	Callus present	bridging callus	remodeled, fracture not visible	cortex	no callus	Callus present	bridging callus	remodeled, fracture not visible
	**Right cortex**	Right cortex score	Right cortex score	Right cortex score	Right cortex score	**Right cortex**	Right cortex score	R1	Right cortex score	Right cortex score
	**Left cortex**	Left cortex score	Left cortex score	Left cortex score	Left cortex score	**Left cortex**	Left cortex score	R2	Left cortex score	Left cortex score
Rabbit 6 Week 7	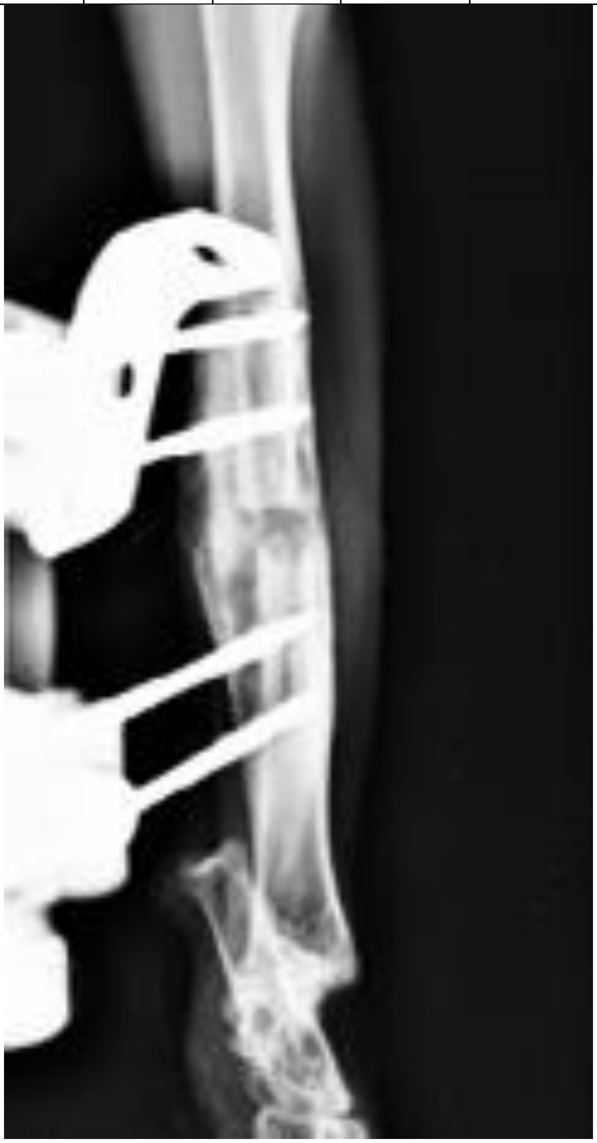					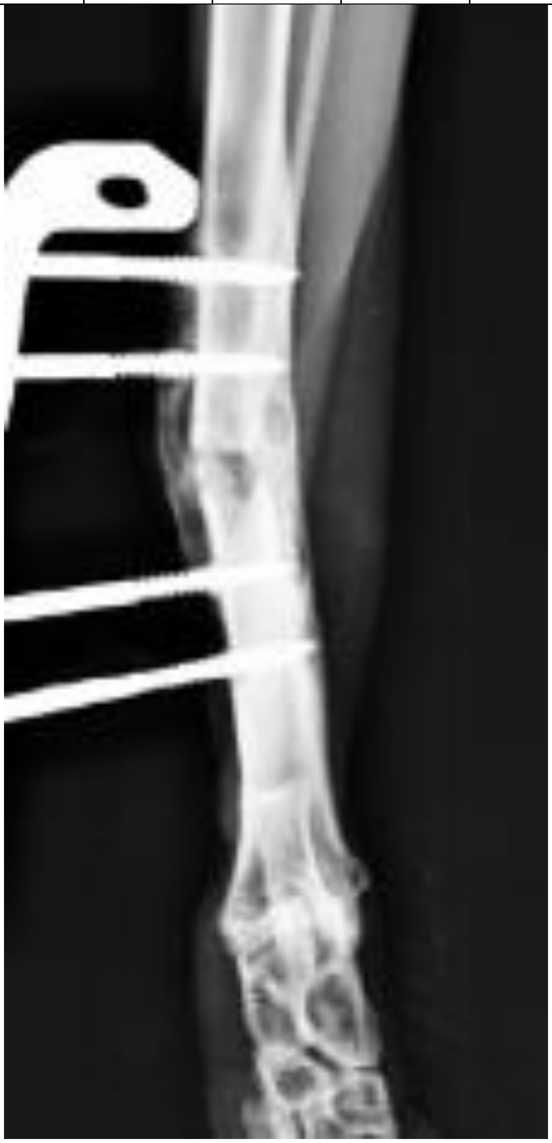				

	Oblique View					Anterior-Posterior View				
	cortex	no callus	Callus present	bridging callus	remodeled, fracture not visible	cortex	no callus	Callus present	bridging callus	remodeled, fracture not visible
	**Right cortex**	Right cortex score	Right cortex score	Right cortex score	Right cortex score	**Right cortex**	Right cortex score	R4	Right cortex score	Right cortex score
	**Left cortex**	Left cortex score	Left cortex score	Left cortex score	Left cortex score	**Left cortex**	Left cortex score	R3	Left cortex score	Left cortex score
Rabbit 7<br>Week 8	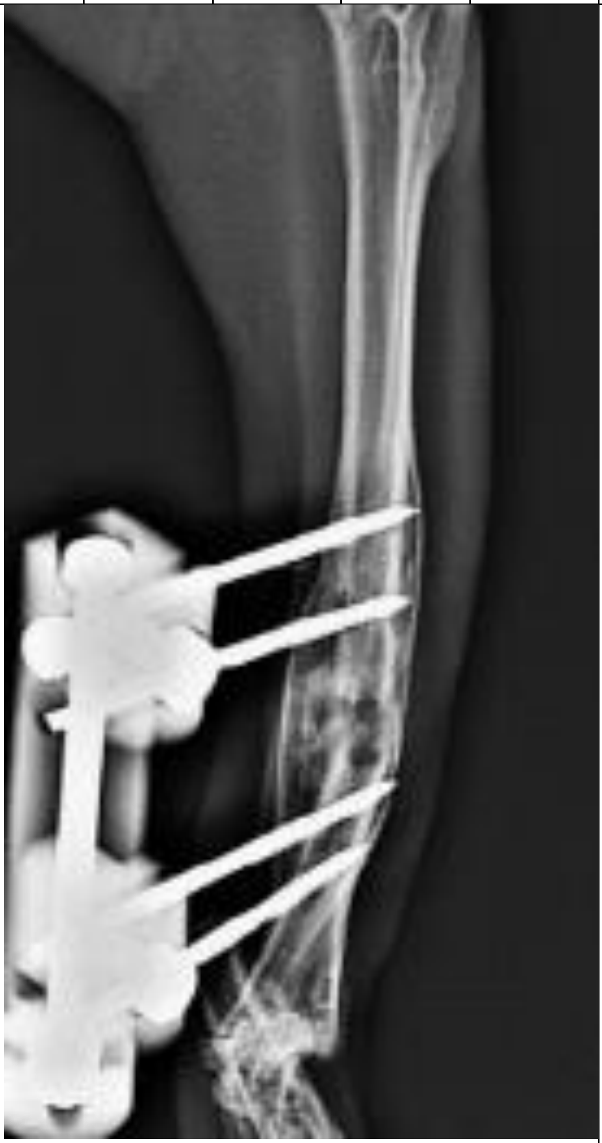					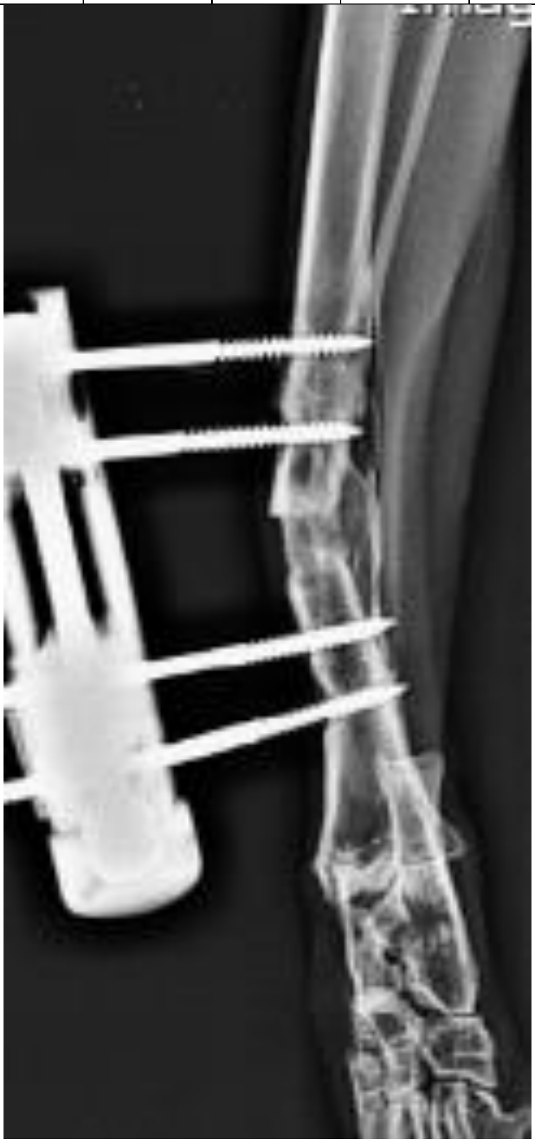				

	Oblique View					Anterior-Posterior View				
	cortex	no callus	Callus present	bridging callus	remodeled, fracture not visible	cortex	no callus	Callus present	bridging callus	remodeled, fracture not visible
	**Right cortex**	Right cortex score	Right cortex score	Right cortex score	Right cortex score	**Right cortex**	Right cortex score	R5	Right cortex score	Right cortex score
	**Left cortex**	Left cortex score	Left cortex score	Left cortex score	Left cortex score	**Left cortex**	Left cortex score	R4	Left cortex score	Left cortex score
Rabbit 8<br>Week 8	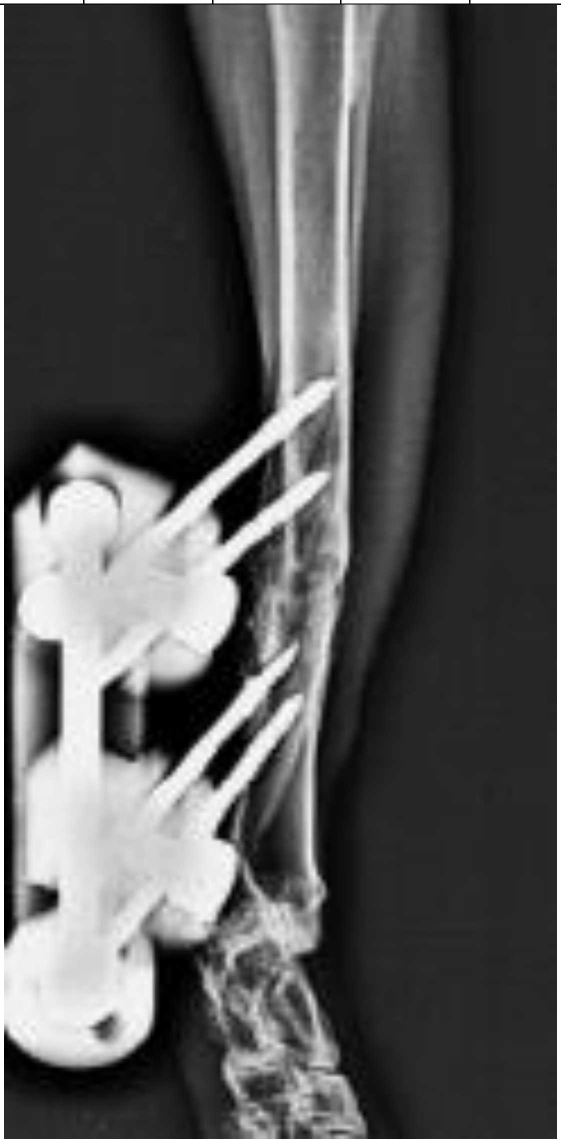					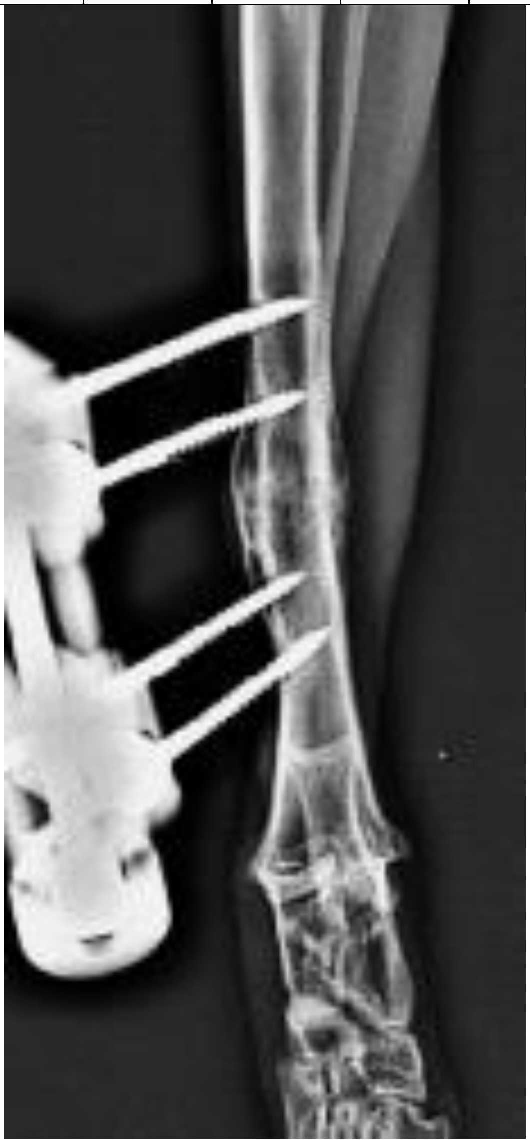				

	Oblique View					Anterior-Posterior View				
	cortex	no callus	Callus present	bridging callus	remodeled, fracture not visible	cortex	no callus	Callus present	bridging callus	remodeled, fracture not visible
	**Right cortex**	Right cortex score	Right cortex score	Right cortex score	Right cortex score	**Right cortex**	Right cortex score	RABBIT 6	Right cortex score	Right cortex score
	**Left cortex**	Left cortex score	Left cortex score	Left cortex score	Left cortex score	**Left cortex**	Left cortex score	R7	Left cortex score	Left cortex score
Rabbit 7 Week 9	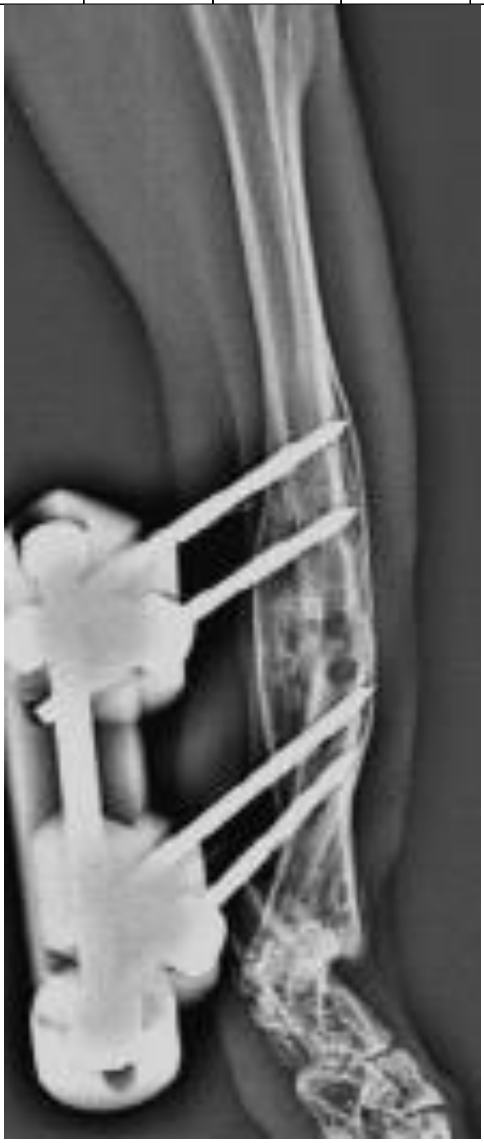					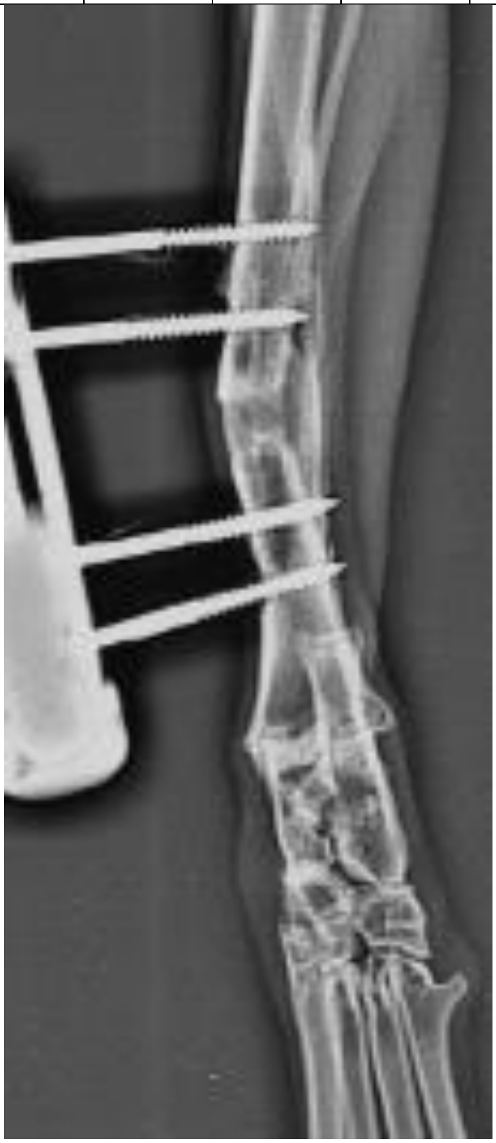				

	Oblique View					Anterior-Posterior View				
	bridging callus	remodeled, fracture not visible	bridging callus	remodeled, fracture not visible	bridging callus	remodeled, fracture not visible	bridging callus	remodeled, fracture not visible	bridging callus	remodeled, fracture not visible
	**Right cortex**	Right cortex score	Right cortex score	Right cortex score	Right cortex score	**Right cortex**	Right cortex score	Right cortex score	Right cortex score	Right cortex score
	**Left cortex**	Left cortex score	Left cortex score	Left cortex score	Left cortex score	**Left cortex**	Left cortex score	Left cortex score	Left cortex score	Left cortex score
Rabbit 8<br>Week 9	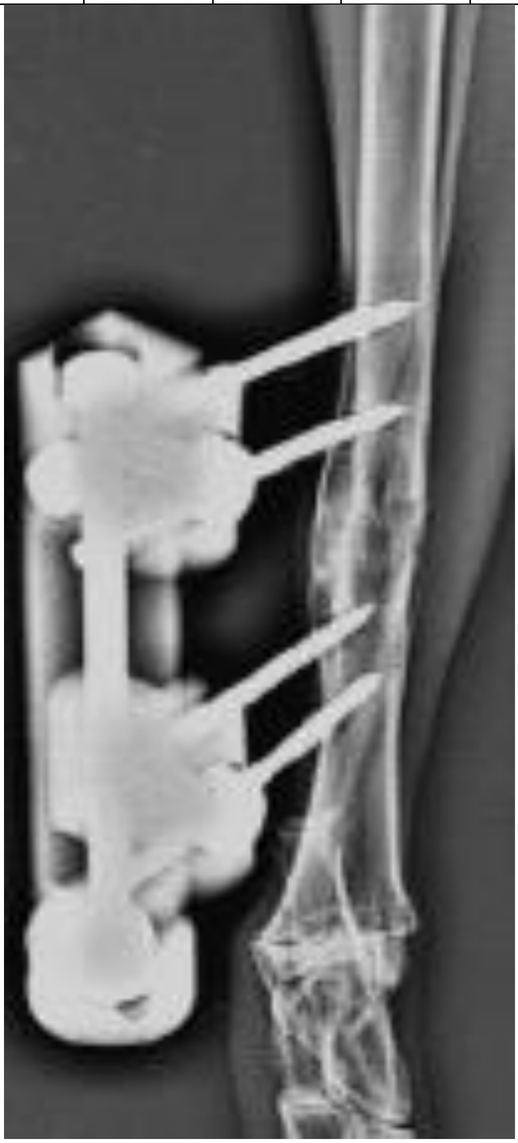					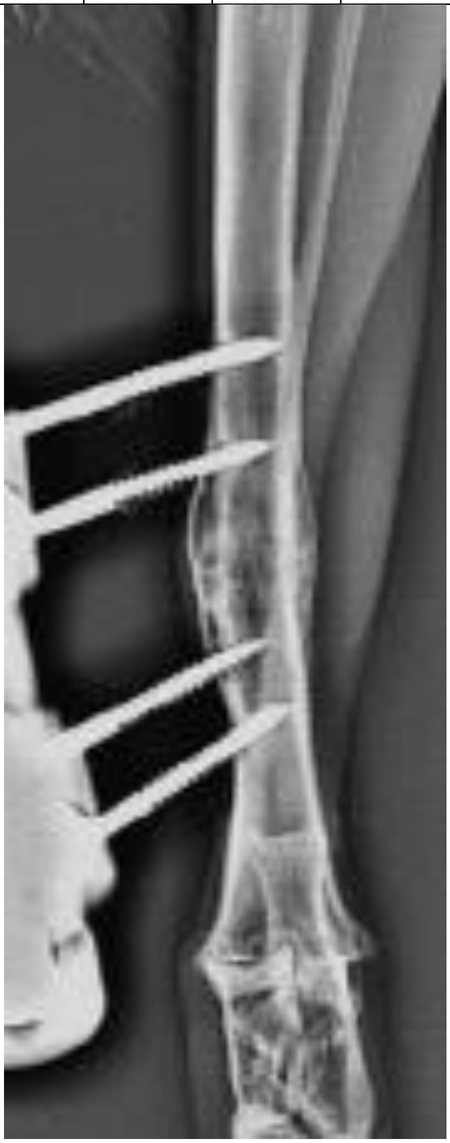				
